# Tau is required for progressive synaptic and memory deficits in a transgenic mouse model of α-synucleinopathy

**DOI:** 10.1007/s00401-019-02032-w

**Published:** 2019-06-06

**Authors:** Balvindar Singh, Ana Covelo, Héctor Martell-Martínez, Carmen Nanclares, Mathew A. Sherman, Emmanuel Okematti, Joyce Meints, Peter J. Teravskis, Christopher Gallardo, Alena V. Savonenko, Michael A. Benneyworth, Sylvain E. Lesné, Dezhi Liao, Alfonso Araque, Michael K. Lee

**Affiliations:** 1 Medical Scientist Training Program, University of Minnesota Medical School, 420 Delaware Street SE, Minneapolis, MN 55455 USA; 2 Graduate Program in Neuroscience, University of Minnesota Medical School, 420 Delaware Street SE, Minneapolis, MN 55455 USA; 3 Department of Neuroscience, University of Minnesota Medical School, 420 Delaware Street SE, Minneapolis, MN 55455 USA; 4 Graduate Program in Pharmacology, University of Minnesota Medical School, 420 Delaware Street SE, Minneapolis, MN 55455 USA; 5 Institute for Translational Neuroscience, University of Minnesota Medical School, 420 Delaware Street SE, Minneapolis, MN 55455 USA; 6 Mouse Behavior Core, University of Minnesota Medical School, 420 Delaware Street SE, Minneapolis, MN 55455 USA; 7 N. Budd Grossman Center for Memory Research and Care, University of Minnesota Medical School, 420 Delaware Street SE, Minneapolis, MN 55455 USA; 8 Geriatric Research Education and Clinical Center, Minneapolis Veterans Affairs Health Care System, University of Minnesota Medical School, 420 Delaware Street SE, Minneapolis, MN 55455 USA; 9 Department of Pathology, Johns Hopkins University School of Medicine, 733 N Broadway, Baltimore, MD 21205 USA

**Keywords:** α-Synuclein, Tau, Parkinson’s disease, Lewy body disease, Dementia, Neuronal plasticity

## Abstract

Parkinson’s disease dementia (PDD) and dementia with Lewy bodies (DLB) are clinically and neuropathologically highly related α-synucleinopathies that collectively constitute the second leading cause of neurodegenerative dementias. Genetic and neuropathological studies directly implicate α-synuclein (αS) abnormalities in PDD and DLB pathogenesis. However, it is currently unknown how αS abnormalities contribute to memory loss, particularly since forebrain neuronal loss in PDD and DLB is less severe than in Alzheimer’s disease. Previously, we found that familial Parkinson’s disease-linked human mutant A53T αS causes aberrant localization of the microtubule-associated protein tau to postsynaptic spines in neurons, leading to postsynaptic deficits. Thus, we directly tested if the synaptic and memory deficits in a mouse model of α-synucleinopathy (TgA53T) are mediated by tau. TgA53T mice exhibit progressive memory deficits associated with postsynaptic deficits in the absence of obvious neuropathological and neurodegenerative changes in the hippocampus. Significantly, removal of endogenous mouse tau expression in TgA53T mice (TgA53T/mTau^−/−^), achieved by mating TgA53T mice to mouse tau-knockout mice, completely ameliorates cognitive dysfunction and concurrent synaptic deficits without affecting αS expression or accumulation of selected toxic αS oligomers. Among the known tau-dependent effects, memory deficits in TgA53T mice were associated with hippocampal circuit remodeling linked to chronic network hyperexcitability. This remodeling was absent in TgA53T/mTau^−/−^ mice, indicating that postsynaptic deficits, aberrant network hyperactivity, and memory deficits are mechanistically linked. Our results directly implicate tau as a mediator of specific human mutant A53T αS-mediated abnormalities related to deficits in hippocampal neurotransmission and suggest a mechanism for memory impairment that occurs as a consequence of synaptic dysfunction rather than synaptic or neuronal loss. We hypothesize that these initial synaptic deficits contribute to network hyperexcitability which, in turn, exacerbate cognitive dysfunction. Our results indicate that these synaptic changes present potential therapeutic targets for amelioration of memory deficits in α-synucleinopathies.

**Electronic supplementary material:**

The online version of this article (10.1007/s00401-019-02032-w) contains supplementary material, which is available to authorized users.

## Introduction

Parkinson’s disease dementia (PDD) and Dementia with Lewy bodies (DLB) are closely related diseases in the α-synucleinopathy family that comprise the second most common neurodegenerative dementia [53]. These and other α-synucleinopathies are characterized by the presence of cytoplasmic inclusions termed Lewy bodies (LB) and Lewy neurites (LN), composed primarily of fibrillar α-synuclein (αS) [105]. In addition to motor dysfunction arising from the loss of dopaminergic neurons in the substantia nigra pars compacta [82], the broad distribution of LB/LN across multiple neuronal populations has led to an understanding in PD that the disease extends beyond the basal ganglia [1, 2]. Dementia in the α-synucleinopathies is hypothesized to be a product of αS abnormalities at cortical and hippocampal synapses. This pathogenic role for αS is supported by genetic and pathological observations: mutations in the *SNCA* gene encoding αS are causative for early onset, familial autosomal dominant forms of PD [48, 92, 93], and LBs and LNs are found in both familial and sporadic cases of PD [104]. While degeneration of cortical and hippocampal neurons is not a significant feature of PDD and DLB [42], cortical and hippocampal αS pathology show significant correlation with dementia [4, 24, 42, 43, 53, 105]. Because αS is a cytosolic protein enriched at presynaptic terminals with established roles as an inhibitor of neurotransmitter release [3, 81] and a presynaptic chaperone [14–16, 18], it is hypothesized that disease-associated αS may cause memory deficits through mechanisms involving presynaptic dysfunction. We recently showed that mutant A53T human αS (hαS^A53T^) expression causes deficits in learning, memory, and synaptic plasticity in mice [110]. Significantly, while we show that presynaptic deficits, characterized by decreased probability of neurotransmitter release, are present in both wild-type and mutant human αS expressing neurons, only hαS^A53T^ expression caused defects in postsynaptic function and synaptic plasticity. Mechanistically, this unique hαS^A53T^-induced postsynaptic dysfunction is mediated through a process involving tau: GSK3β-dependent tau phosphorylation, subsequent tau missorting to dendritic spines, and calcineurin-dependent AMPA receptor (AMPAR) internalization. These deficits in neurotransmission appear in the absence of overt neuropathology, suggesting that neuronal dysfunction is not a consequence of neurotoxicity and neurodegeneration.

In this study, we sought to extend our prior findings by mechanistically connecting our in vitro studies with the memory deficits in vivo. To accomplish this, we directly tested whether tau expression was required for αS-induced cellular, physiological, and behavioral deficits in the TgA53T mouse model of α-synucleinopathy. We show that TgA53T mice exhibit progressive memory deficits associated with the presence of postsynaptic, but not presynaptic, deficits. More important, we demonstrate that loss of tau completely reversed the onset of memory deficits in multiple experimental paradigms and that tau is required for αS-mediated neurophysiological deficits, including postsynaptic dysfunction, impairments in glutamatergic neurotransmission, and short- and long-term plasticity. Significantly, these parameters are independent of any αS pathology or neurodegenerative changes. Finally, the onset of neurophysiological and memory deficits coincides with the onset of seizure-like network hyperactivity. We propose that early tau-dependent postsynaptic deficits caused by mutant αS are mechanistically linked to the onset of network abnormalities and memory deficits. Our results provide novel insights on how pathological αS precipitates impairments in neurotransmission and memory loss and may inform the development of new therapeutic approaches for PDD and DLB.

## Materials and methods

All animal studies were performed in accordance with the NIH guidelines for the use of animals in research and approved by the Institutional Animal Care and Use Committee at the University of Minnesota. Experimental group sizes (*n*) are reported in each figure.

### Transgenic A53T α-synuclein mutant and tau-knockout mice

We bred four key genotypes for this study. TgA53T animals contain a transgene expressing the human mutant A53T α-synuclein (αS, hαS^A53T^: line G2-3). All mice were maintained in the C57BL/6J background strain (stock 0006644, Jackson Labs; Bar Harbor, ME, USA). TgA30P animals express human mutant A30P αS (hαS^A30P^: line O2,TgA30P) at similar levels to TgA53T, and TgWT animals express human wild-type αS (hαS^WT^: line I2-2, TgWT), but at levels lower to TgA53T and TgA30P. Transgene expression for all animals is controlled by the mouse prion promoter (MoPrP) [65]. TgA53T, TgA53T/mTau^−/−^, TgA30P, and TgWT were all heterozygous for their respective hαS transgene. Non-transgenic (nTg) controls came from within these litters. To generate transgenic animals expressing hαS^A53T^ (TgA53T) and lacking endogenous mouse tau (mTau^−/−^), TgA53T males were bred to Mapt^tm1(EGFP)Klt^/J females (stock 004779, Jackson Labs) [113] in successive generations to generate offspring that lacked endogenous mouse tau expression and with, or without, hαS^A53T^ transgene expression. Transgenic progenies were identified by PCR analysis of tail DNA as previously described [65]. Because all mice were maintained on the C57BL/6J background, we were able to generate TgA53T and nTg cohorts separate from the TgA53T/mTau^−/−^ and mTau^−/−^ cohorts. This approach was used, because the use of heterozygous mice for breeding leads to a very low yield of animals with the experimentally desired genotype (25%), increased heterogeneity between littermates, and more importantly, a larger spread in birthdates and consequently ages of mice, introducing potential methodological variations. In addition, this low yield of experimentally desired mice would translate into a large population of unused, “wasted”, animals, something we consciously worked towards avoiding. Generating TgA53T/mTau^+/+^ (TgA53T) and TgA53T/mTau^−/−^ cohorts via independent homozygous matings produced progeny within a tighter age range and allowed us to use 100% of the offspring for experiments.

The TgA53T (G2–3) mouse model exhibits an adult-onset hyperactive locomotor phenotype [116] prior to the onset of motor dysfunction. This motor dysfunction phenotype rapidly progresses from slight ataxic signs (gait and balance abnormalities) to complete paralysis within 2–3 weeks [65]. This pattern of motor deficit is also observed in another transgenic mouse model expressing hαS^A53T^ under control of the MoPrP promoter (M83) as rotarod performance declines only following sudden and dramatic disease onset [35]. Since the initial characterization of TgA53T mice, the average age of disease onset has drifted from approximately 10 months of age to approximately 14 months of age (Lab of Michael K. Lee, unpublished data). Neuropathologically, the onset of motor symptoms in MoPrP-hαS^A53T^ transgenic mice (G2–3 and M83) is associated with the presence of αS pathology in subcortical areas (midbrain, brain stem, and spinal cord) but not in forebrain regions including the hippocampus and amygdala [35, 65]. Any animals showing overt motor symptoms were excluded from further analysis.

Male mice were exclusively used for all behavior studies. For the electrophysiological, biochemical, and immunohistochemical analyses, both males and females were used. In these studies, efforts were made to use equal numbers of mice from each sex within each genotype cohort.

All mice in the colony were kept under specific pathogen-free (SPF) conditions in a 14 h (h) light/10 h dark cycle, and had free access to food and water. All housing and experimental protocols involving mice were conducted with strict adherence to the National Institutes of Health (NIH) Animal Care and Guidelines and were reviewed and approved by the Institutional Animal Care and Use Committee (IACUC) at the University of Minnesota.

### Dissociated neuronal culture electrophysiology

Dissociated neuronal cultures were established from harvested from mouse hippocampi postnatal day 0 or 1 (P0–P1) as previously described [110] with modifications. Briefly, mouse hippocampi were dissected out and stored individually by pup in Hibernate A media (Brain Bits; Springfield, IL, USA) at 4 °C. While stored, each pup from the cultured litter was genotyped. Cerebellar tissue was used for genomic DNA isolation via QuickExtract DNA (Epicentre-Lucigen; Madison, WI, USA) and genotyping of pups was performed immediately after hippocampal isolation. Following genotype identification, hippocampi of identical genotypes were pooled, dissociated, and plated on onto 35 mm μ-dishes containing a poly-d-lysine and laminin-coated polymer coverslip (Ibidi; Fitchburg, WI, USA). 24 h after initial plating, media were removed and NbActiv4 growth media were added (Brain Bits). NbActiv4 media were refreshed every 3–4 days by removing 1 mL media in the dishes and adding 1 mL fresh media. For all procedures and media exchanges, solutions were equilibrated in a tissue culture incubator (37 °C, 5% CO_2_) for at least 2 h prior to use. The age of in vitro dissociated cultured hippocampal neurons began with the day of initial plating, and each day that followed was counted as 1 day in vitro (DIV). All experiments were performed on neurons from at least three independent cultures with a minimum of five animals per culture. Miniature excitatory postsynaptic currents (mEPSC) were recorded from cultured dissociated mouse hippocampal neurons at 21–25 DIV with a glass pipette (resistance of ~ 5 MΩ) as previously described [110]. Recordings ranged from 3 to 15 min and stable traces longer than 1 min in duration were analyzed. All mEPSCs > 2 pA were manually counted with MiniAnalysis (Synaptosoft Inc; Fort Lee, NJ, USA). Each mEPSC event was visually inspected and only events with a distinctly fast-rising phase and a slow-decaying phase were accepted. Relative cumulative frequencies were derived from individual events and the averaged parameters from each neuron were treated as single samples in any further statistical analyses.

### Acute hippocampal slice electrophysiology

Acute coronal hippocampal slices (approximately 350 μm thick) were obtained from two age windows of mice (2–3 months and 5–6 months) from nTg, TgA53T, TgA53T/mTau^−/−^, and mTau^−/−^ animals utilizing well-established methods [110].

Briefly, slices were incubated in ACSF (containing in mM: NaCl 124, KCl 5, NaH_2_PO_4_ 1.25, MgSO_4_ 2, NaHCO_3_ 26, CaCl_2_ 2, glucose 10; gassed with 95% O_2_, 5% CO_2_, and maintained between pH 7.3 and 7.4) at room temperature for at least 1 h before use. For studies, slices were transferred to an immersion-recording chamber, superfused at 2 mL/min with oxygenated ACSF, and visualized under an Olympus BX50WI microscope (Olympus Optical; Japan). To study excitatory postsynaptic currents (EPSCs), picrotoxin (50 μM) and CGP54626 (1 μM) were added to the solution to block GABA-A and GABA-B receptors, respectively. Whole-cell electrophysiological recordings were obtained from hippocampal CA1 pyramidal neurons using patch electrodes (3–10 MΩ) filled with an internal solution containing in mM: cesium gluconate 117, HEPES 20, EGTA 0.4, NaCl 2.8, TEA-Cl 5, ATP 2, GTP 0.3, and kept at pH 7.3. Recordings were obtained with PC-ONE amplifiers (Dagan Instruments; Minneapolis, MN, USA). Membrane potentials were held at − 70 mV. Signals were filtered at 1 kHz, acquired at a 10 kHz sampling rate, and fed to a Digidata 1440A digitizer (Molecular Devices; San Jose, CA, USA). pCLAMP 10.4 (Axon Instruments, Molecular Devices; San Jose, CA, USA) was used for stimulus generation, data display, data acquisition, and data storage. To record evoked EPSCs, theta capillaries filled with ACSF were used for bipolar stimulation and placed in the stratum radiatum to stimulate Schaffer collaterals. Input–output curves of EPSCs were made by increasing stimulus intensities from 0 to 100 μA. Paired-pulse facilitation was done by applying paired pulses [2 ms (ms) duration] with 25, 50, 75, 100, 200, 300, and 500 ms inter-pulse intervals. The paired-pulse ratio was calculated by dividing the amplitude of the second EPSC by the first (PPR = EPSC-2/EPSC-1). Synaptic fatigue was assessed by applying 15 consecutive stimuli in 25 ms intervals. AMPA currents were obtained at a holding potential of − 70 mV. NMDA currents were obtained at a holding potential of + 30 mV. To ascertain the AMPA-to-NMDA receptor current ratio, the NMDA component was measured 50 ms after the stimulus when the AMPA component had decayed. Recordings of miniature EPSCs (mEPSCs; *V*_h_ = –70 mV) were made in the presence of tetrodotoxin (TTX; 1 μM) in addition to the respective solution. For long-term potentiation (LTP), CA1 pyramidal neurons first underwent a baseline recording of 10 min followed by tetanic stimulation in the Schaffer collaterals (4 trains at 100 Hz for 1 s; 30 s intervals). After LTP induction, neurons were recorded for 50 min. The presence of LTP was determined by comparing the average EPSC amplitudes from last 5 min of pre-stimulus baseline recording to the average EPSC amplitudes from the last 5 min post-stimulus recordings. All experiments were performed at room temperature.

### Behavioral testing

Behavioral testing was performed with the support and guidance of the Mouse Behavior Core at the University of Minnesota. ANY-maze software (Stoelting Co.; Wood Dale, IL, USA) was used in conjunction with paired video cameras to track and record animal movements during all behavioral testing and all subsequent analyses. Behavior experiments, unless dictated by testing protocols, were not performed on consecutive days, giving animals at least 2 days to rest between experiments. Mice were also habituated to handling prior to testing. Animals from all genotypes were tested together. Each behavioral age point came from a separate cohort. In addition, all groups tested, including those at different age points, went through the battery of tests in the same order.

### Open field

Locomotor activity was assessed using open-field testing. Animals were placed individually into a square 40 cm × 40 cm arena with white, opaque walls and flooring, lit to 200 lx. Movement was videorecorded for 60 min and began immediately after placing the animal in the open-field testing chamber. After the test, animals were returned to their home cage.

### Barnes maze

Spatial learning and memory were assessed via Barnes maze (San Diego Instruments; San Diego, CA, USA), following established protocols [8, 108]. The maze itself consists of a white plastic circular top with 20, evenly sized and evenly spaced holes around the perimeter. Spatial cues were placed on all four walls of the behavioral testing room, lit to 300 lx during testing. Mice were acclimatized to the testing room for 30 min at the beginning of each training session. Training days consisted of 4 trials per day for 4 days. Training trials ended with the subject climbed into the escape box within the goal quadrant located under 1 of the holes or when the maximum trial duration of 180 s was reached. Upon entering the escape box during training, room lights turned off and animals remained in the escape box for 60 s before returning to their home cage in the testing room to await the next trial. While the platform was rotated between each trial on each day (to obscure the impact any animal scents or non-spatial cues on the maze), the location of the escape box and goal quadrant relative to the room remained constant during all training trials and days. Subjects were run in small groups of six mice or less, so that no more than 20 min passed between trials for a given animal during training. On the day following the last training trial, memory was assessed in single 90 s probe trial tests, where the target escape box in the goal quadrant was replaced with a false box cover identical to the other 19 holes, and the exploration pattern of each subject was examined.

### Y maze

Short-term spatial learning and memory was assessed via Y maze. Animals underwent 30 min acclimation periods in the testing room prior to testing. Cues were visible on walls of the testing room as well as within the maze, at the end of each arm (extra-maze and intra-maze, respectively). The Y maze was constructed out of opaque white plastic and located at the center of the testing room lit to 150 lx. During the first of two phases, only two of the three arms were available for exploring during the 10 min learning trial: the start arm and the familiar arm. For all trials, animals were placed at the end of the start arm, furthest point from center. During the 60 min inter-trial interval between learning and recognition, animals returned to their home cage, but remained in testing room. The 5 min recognition trial was then performed, with all three arms were available for exploration: start, familiar, and novel. The Y maze was also cleaned between each trial/animal to avoid confounds due to scent.

### Context fear conditioning

Context fear conditioning was conducted using the Near-Infrared (NIR) Video Fear Conditioning package for mice (Med Associates, Inc; Fairfax, VT, USA). A context conditioning environment (specific olfactory, tactile, and visual elements) is paired with foot shocks on the first day: a 10 min context conditioning trial was first performed in the NIR fear conditioning chamber: electrified metal bars on the floor, providing 0.7 V foot shocks for 2 s 5 times per trial at variable intervals averaging 90 s between shocks, squared off corners and metal walls, and a 33% Simple Green solution for scent. 24 h following conditioning animals were underwent the context memory trial, where freezing was assessed for 3 min with identical chamber conditions to the conditioning trial except no foot shocks (similar). If the animal successfully learns the context–shock association, re-exposure to the context environment 24 h post-conditioning, the “similar” setting, will elicit fear in the animal placed in that environment. 2 h after the similar trial, mice were placed in the chamber for the novel context trial (Novel), white, solid plastic flooring and curved walls with a stripe design inside, and a 0.5% vanilla solution for scent, to show the context specificity of the fear memory.

### Western blot analyses

#### Chemiluminescence western blotting

At predefined age points, mice were harvested and brain regions (cortices and hippocampi, in particular) were dissected out and stored at − 80 °C. To extract proteins, cortical and hippocampal samples from − 80 °C were first thawed at − 20 °C overnight before being weighed and homogenized via Dounce homogenization in 10 volumes of TNE solution in mM: Tris–HCl 50, NaCl 150, and EDTA 5. Following homogenization, an equal volume of Complete TNE was added: TNE plus 0.5% NP40, 0.5% DOC, 1% SDS, and HALT protease and phosphatase inhibitors (Thermo Fisher Scientific; Waltham, MA, USA). Samples were then spun down at 16,000 g for 90 min at 4 °C. Supernatant was collected and subjected to immunodepletion via serial incubations with Protein A and G Mag Sepharose beads (GE Life Science; Pittsburgh, PA, USA) rocking at 4 °C for 60 min. Protein concentration was assessed via BCA assay (Pierce, Thermo; Rockford, IL, USA). Samples were prepared to equal concentration in reducing, SDS sample, Laemmli buffer (Boston BioProducts; Ashland, MA, USA). For western blot analysis, protein lysates were run on Criterion™ TGX™ gels (Bio-Rad; Hercules, CA, USA) and transferred onto nitrocellulose membranes. Membranes were then blocked using 5% non-fat dry milk in tris-buffered saline (TBS) with 0.1% Tween 20 (TBSTw) for 1 h at room temperature (RT). Primary antibodies were diluted in OneBlock solution (Genesee Scientific; San Diego, CA, USA) and incubated on membranes overnight at 4 °C. Horseradish peroxidase (HRP)-conjugated secondary antibodies (Invitrogen; Carlsbad, CA, USA) were diluted in 5% non-fat dry milk in TBSTw and incubated on membranes 1 h at RT. Membranes were then developed using chemiluminescent substrates (Bio-Rad and Thermo) and the ImageQuant LAS 4000 detection system (GE Life Sciences). Densitometry on western blot images was subsequently analyzed using the ImageQuant TL 8.1 software (GE Life Sciences).

#### Fluorescence western blotting

SDS-PAGE was performed on precast 10.5–14% Criterion™ Tris-Tricine gels, 10.5–14% or 4–10.5% Tris–HCl gels (Bio-Rad). Protein levels were normalized using 10–100 μg of protein per sample (depending on the targeted protein). The samples were resuspended with 4 × Tricine loading buffer and boiled for 5 min before loading. Proteins were transferred onto 0.2-μm nitrocellulose membrane (Bio-Rad) following electrophoresis. Membranes were blocked in 5% BSA (Sigma; St. Louis, MO, USA) in TBSTw for 1–2 h at room temperature and probed with the appropriate antisera/antibodies diluted in 5% BSA in TBSTw. Primary antibodies were probed with secondary antibodies conjugated with infrared dyes (LI-COR Biosciences; Lincoln, NE, USA). Densitometry analyses were performed using the Odyssey software (LI-COR Biosciences). Normalization was performed against GAPDH. Quantification by software analysis was performed as described previously [5, 56, 59–61, 103].

### Dot blot analyses

Protein isolates were obtained from samples at the same time that they were being prepared for western blotting. Samples diluted to identical concentrations (1 μg/μL) and 2 μg of sample were adsorbed onto a nitrocellulose membrane and air-dried for 30 min. Following activation in 10% methanol (in TBS), membranes were blocked in TBS containing 5% bovine serum albumin (BSA) for 30 min at room temperature (RT). Samples were then incubated in primary antibody overnight at 4 °C in 5% BSA in TBS. Following primary antibody incubation and TBS washes, secondary incubation was performed for 1 h at RT in the dark in 5% BSA in TBS with IRDye secondary antibodies (anti-mouse IgG-IR800 at 1:100,000 and anti-rabbit IgG-IR680 at 1:150,000) (LI-COR Biosciences). Membranes were then washed, imaged, and analyzed using the LI-COR Odyssey-imaging system.

### Insoluble and soluble protein extraction

An equal volume of 2% Triton™ X-100 (Sigma-Aldrich; St. Louis, MO, USA) in TNE was added to the Dounce homogenized lysate in TNE, mixed, and then sonicated for 30 s at 4 °C. Samples were then spun down at 20,000 g for 60 min at 4 °C. The supernatant from this spin was saved as the soluble fraction. The pellet was saved as the insoluble fraction, washed in 1% Triton™ X-100 in TNE and spun down again. Complete TNE was added to both soluble and insoluble fractions. Fractions were then sonicated, boiled for 10 min at 95 °C, and spun down at 16,000*g* for 15 min at 4 °C, and then, the supernatant from each fraction was saved. Protein concentration was assessed via BCA assay (Pierce, Thermo; Rockford, IL, USA). Samples were then prepared for western blotting identically to the “Chemiluminescent Western Blotting” protocol. After transfer, membranes were stained with 0.1% Ponceau S solution (Sigma-Aldrich) to confirm equal protein loading and transfer. Samples then underwent primary and secondary antibody incubation and chemiluminescent detection following “Chemiluminescent Western Blotting” methods.

### Immunohistochemical analyses

At predefined age points, mice were anesthetized with isoflurane and euthanized via transcardial perfusion with saline. Animals were first perfused with ice cold K-free PBS, containing in mM: NaCl 13, Na_2_HPO_4_·2H_2_O 0.7, and NaH_2_PO_4_·2H_2_O 0.3, and adjusted to pH 7.2. Following saline perfusion, animals were then perfuse-fixed with 4% paraformaldehyde (PFA). Brains were then isolated and stored in 4% PFA at 4 °C for an additional 48 h before being transferred to a 30% sucrose solution in PBS prior to slicing. After a minimum of 72 h in sucrose solution, brains were sliced coronally at a thickness of 30 μM via HM 450 sliding microtome (Thermo Fisher Scientific) and stored in a cryoprotectant solution (30% sucrose and 30% ethylene glycol in PBS) prior to immunostaining. Immunofluorescent staining was preformed utilizing the Pelco BioWave Plus (Ted Pella; Redding, CA, USA) and mounted on slides for imaging. Prior to staining sections were rinsed in TBS, permeabilized with 0.1% Triton X-100 in TBS and blocked in 50% Background Sniper solution in TBS (Biocare Medical; Pacheco, CA, USA). Primary and secondary antibodies were diluted to target concentrations in 5% Background Sniper (Biocare Medical) in TBSTw and allowed to incubate on sections in the Pelco BioWave for 1.5 h each. Alexa Fluor (Abcam; Cambridge, MA, USA) or Alexa Fluor Plus (Thermo Fisher Scientific) secondary antibodies were used as dictated by primary antibody host. If required, sections were then 4′,6-diamidino-2-phenylindole (DAPI)-counterstained. All sections were treated with TrueVIEW™ (Vector Laboratories; Burlingame, CA, USA) for autofluorescence quenching and mounted on slides for future imaging. Confocal microscopy images were acquired with the Nikon C2 Confocal Microscope System (Nikon; Melville, NY, USA). Imaging processing and analysis were performed using the NIS-Elements software (Nikon). Staining, imaging, and analysis were performed in collaboration with the University of Minnesota’s University Imaging Centers on the Twin Cities campus. Analysis and quantification of biomarkers of neuronal network hyperexcitability [c-Fos, neuropeptide Y (NPY), and calbindin] were performed as described previous [89].

### Antibodies

A detailed list of all antibodies used for experiments and studies here can be found in Suppl. Table 1 (Online Resources).

### Experimental design and statistical analyses

All statistical analyses were performed in Prism 8.1.0 (GraphPad Software; San Diego, CA, USA). Data visualization and presentation were performed via Prism (GraphPad) and JMP 14 (SAS; Cary, NC, USA). For parametric data: one-, two-, and three-way ANOVAs were utilized for one, two, and three variable analyses on multiple groups, respectively; *t* tests were also performed when analyzing only two groups. For nonparametric data, Kruskal–Wallis one-way ANOVA for three or more groups and Mann–Whitney test for two groups. Posthoc analyses were performed on all data that were significantly different. Welch’s correction, Sidak’s posthoc analysis, and Tukey’s posthoc analyses were used due to considerations for within-group variances and sample size. The Geisser–Greenhouse correction was used for two- and three-way repeated measures ANOVAs due to considerations for sphericity. For all, statistical significance was set for *α* = 0.05. Data representations are described in figure legends.

## Results

### Mutant α-synuclein-dependent cognitive dysfunction in spatial learning and memory is both progressive and tau-dependent

Several α-synuclein (αS) transgenic mouse models display memory deficits [19, 67, 68, 70], including the model expressing human mutant A53T αS (TgA53T) [19, 67, 68, 90, 110]. However, the mechanistic basis for cognitive impairment in these mouse models is poorly defined. Previously, we established a novel connection between human mutant A53T αS (hαS^A53T^) and postsynaptic impairments in AMPAR function that require tau phosphorylation and mislocalization to dendritic spines [110]. Because these glutamatergic postsynaptic impairments are linked to cognitive dysfunction [47], we hypothesized that cognitive deficits in TgA53T mice are tau-dependent. To directly test the requirement of tau in mediating αS-induced memory loss, we generated TgA53T mice lacking endogenous mouse tau expression by breeding TgA53T mice to the mouse tau-knockout mice (mTau^−/−^) [113]. The four genotypes (TgA53T, TgA53T/mTau^−/−^, mTau^−/−^, and non-transgenic (nTg) littermate controls) appear to develop and age normally for the duration of the study.

Previously, we showed that TgA53T mice exhibit spatial memory deficits in the Barnes maze (BM) [8, 108] at 12 months of age [110]; however, it is unknown if general overexpression of hαS also leads to memory deficits. To confirm that only TgA53T mice exhibit memory deficits in our experimental conditions, we examined whether TgWT (hαS^WT^) and TgA30P (hαS^A30P^) mice in our colony exhibit deficits in spatial learning and memory at older ages. TgA53T and TgA30P mice have similar human mutant αS transgene expression levels, while TgWT mice display lower human αS transgene expression by comparison [110]. We previously showed that TgWT and TgA30P neurons exhibit presynaptic deficits as indicated by reductions in the probability of neurotransmitter release, but intact postsynaptic function and long-term potentiation (LTP) [110]. Thus, we employed these models to directly test whether presynaptic deficits alone are sufficient to cause impairments in learning and memory. Spatial learning and memory analysis of 12-month-old TgWT, TgA30P, and nTg littermates using the BM paradigm shows that all groups of mice are able to learn and retain goal quadrant and escape box location (Suppl. Figure 1b–d, Online Resources). Taken together, our findings show that αS-dependent presynaptic impairments alone are not sufficient to cause memory deficits in mouse models of α-synucleinopathy under our experimental conditions and that memory deficits in TgA53T mice involve both pre- and postsynaptic abnormalities.

**Fig. 1:**
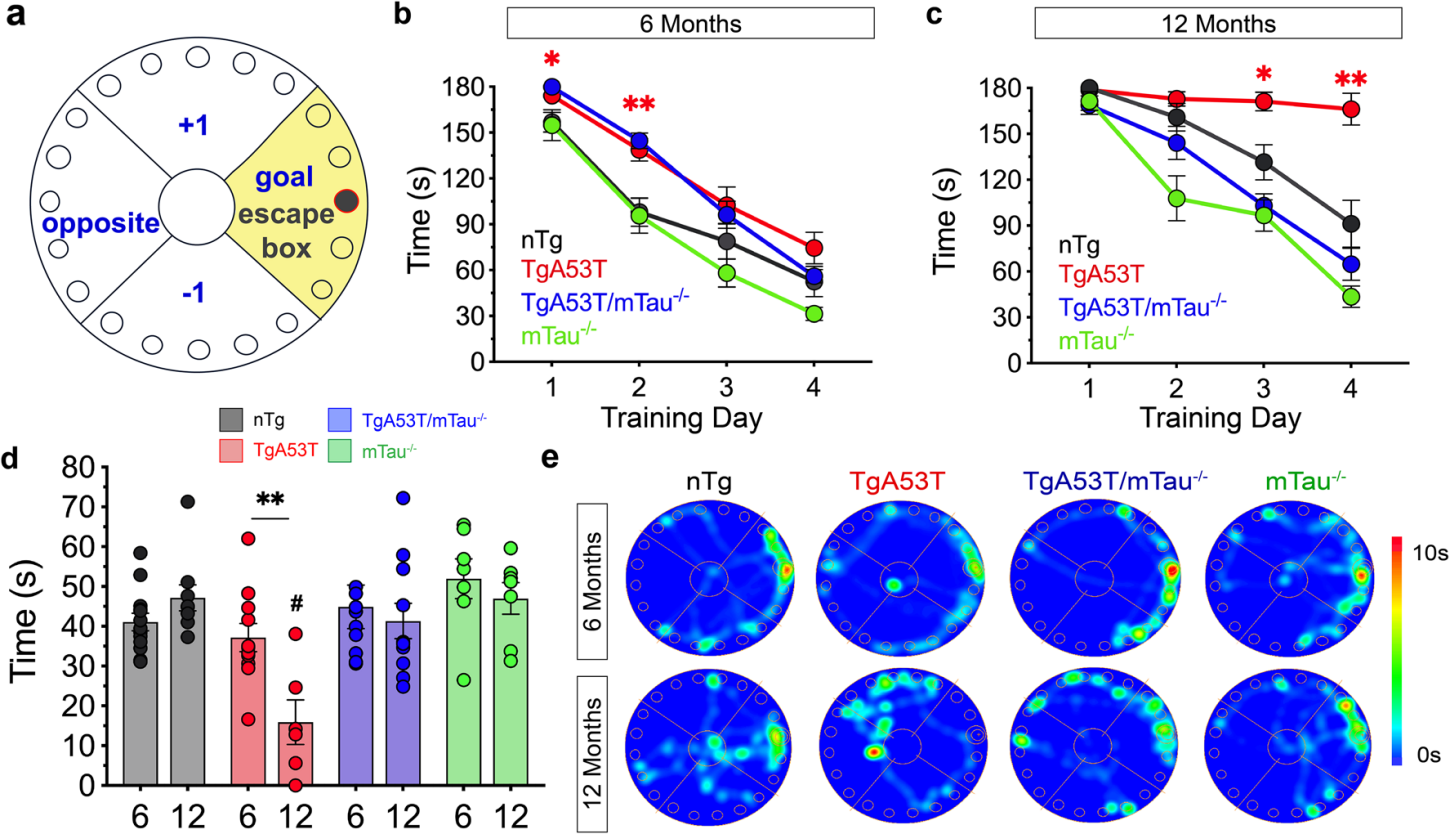
Mutant A53T αS-driven deficits in spatial learning and memory are progressive and tau-dependent. Average duration of training trials per group for each of the 4 training days during the Barnes Maze (BM) when tested at either 6 (**a**) or 12 months of age (6M or 12M) (**b**). 6M training: three-way repeated measures ANOVA with Geisser–Greenhouse correction and Tukey’s posthoc analysis revealed a significant effect of training day [*F*_(2.448,105.3_) = 190.7, *p* < 0.0001], significant effect of hαS^A53T^ genotype [*F*_(1,43_) = 18.63, *p* < 0.0001], no significant effect of mTau^−/−^ genotype [*F*_(1,43_) = 190.7, *p* = 0.2926], and no significant training day*hαS^A53T^*mTau^−/−^ interaction [*F*_(3,129)_ = 0.1160 *p* = 0.9506]. 12M training: three-way repeated measures ANOVA with Geisser–Greenhouse correction and Tukey’s posthoc analysis revealed a significant effects of training day [*F*_(2.192,63.56)_ = 62.15, *p* < 0.0001], hαS^A53T^ genotype [*F*_(1,29)_ = 13.78, *p* = 0.0009], and mTau^−/−^ genotype [*F*_(1,29)_ = 49.05, *p* < 0.0001], and no significant training day*hαS^A53T^*mTau^−/−^ interaction [*F*_(3,87)_ = 3.734, *p* = 0.0141]. **c** BM diagram for testing and probe trial, with yellow shading indicating goal quadrant and dark grey showing escape box location in that quadrant. **d** Time spent in goal quadrant during probe trial for 6M or 12M mice. To compare within genotype between 6 and 12M: unpaired *t* test with Welch’s correction; TgA53T: *t* = 3.369 and *df* = 15 (**p = 0.0042). To compare between genotypes at 6M or 12M: one-way ANOVA with Tukey’s posthoc analysis. 12M group probe test: *F*_(3,29)_ = 9.216 (^#^*p* = 0.0002). 6M: *n*_nTg_ = 13; *n*_TgA53T_ = 11; *n*_TgA53T/mTau_^−/−^ = 10; *n*_mTau_^−/−^ = 8. 12M: *n*_nTg_ = 9; *n*_TgA53T_ = 7; *n*_TgA53T/mTau_^−/−^ = 11; *n*_mTau_^−/−^ = 8. **e** Probe test BM occupancy heat maps during probe test obtained by averaging the location of all animals in each genotype and cohort. Orientation of BM is shown in **c**. In all figures: (1) the color code is: nTg (black), TgA53T (red), TgA53T/mTau^−/−^ (blue), and mTau^−/−^ (green); (2) the data are expressed as mean ± standard error of the mean (SEM); and (3) *p < 0.05, **p < 0.01, ***p < 0.001, and ****p < 0.0001, unless stated otherwise

To better define memory deficits in TgA53T mice, we assessed the progressive nature these deficits hippocampal-dependent spatial learning and memory via BM and whether endogenous tau was required for cognitive deficits in TgA53T mice. At 6 months of age, TgA53T mice exhibit a slight delay in acquisition compared to the nTg land mTau^−/−^ controls (Fig. 1a), mitigated by extra training, as they successfully learn the location of the escape hole in the goal quadrant in a timing similar to controls by training day 4 (Fig. 1a). Consistent with the previous data, 12-month-old TgA53T mice display severe impairments in the ability to learn the BM task, as they failed to display improved performance over consecutive training days (Fig. 1b). Significantly, the loss of tau completely reverses the hαS^A53T^-mediated learning deficit exhibited by TgA53T mice, as TgA53T/mTau^−/−^ showed normal learning during training in the acquisition phase.

In the ensuing probe trial, a putative measure for spatial learning and memory retention, all 6-month-old animals, including TgA53T, spent significantly more time in the goal quadrant compared to other quadrants in the maze (Fig. 1d, e; Suppl. Figure 2a, f, Online Resources), indicating that TgA53T mice have normal long-term spatial memory at 6 months of age. By 12 months, TgA53T mice did not show evidence of spatial memory retention during the probe trial, consistent with impaired memory acquisition (Fig. 1d, e; Suppl. Figure 2b, f, Online Resources). The memory deficits in the 12-month-old TgA53T mice is independent of motor deficits, as the total distance traveled during the probe test was comparable between groups at both 6 and 12 months of age (Suppl. Figure 2c, d, Online Resources).

Consistent with the normal learning and acquisition exhibited by TgA53T/mTau^−/−^ mice, loss of tau expression completely rescues TgA53T-associated memory deficits as 12-month-old TgA53T/mTau^−/−^ effectively learn and recall escape hole location within the goal quadrant. Collectively, these results demonstrate that tau expression is required for the progressive spatial learning and memory deficits in the TgA53T model.

### TgA53T mice exhibit deficits in multiple memory modalities

**Fig. 2:**
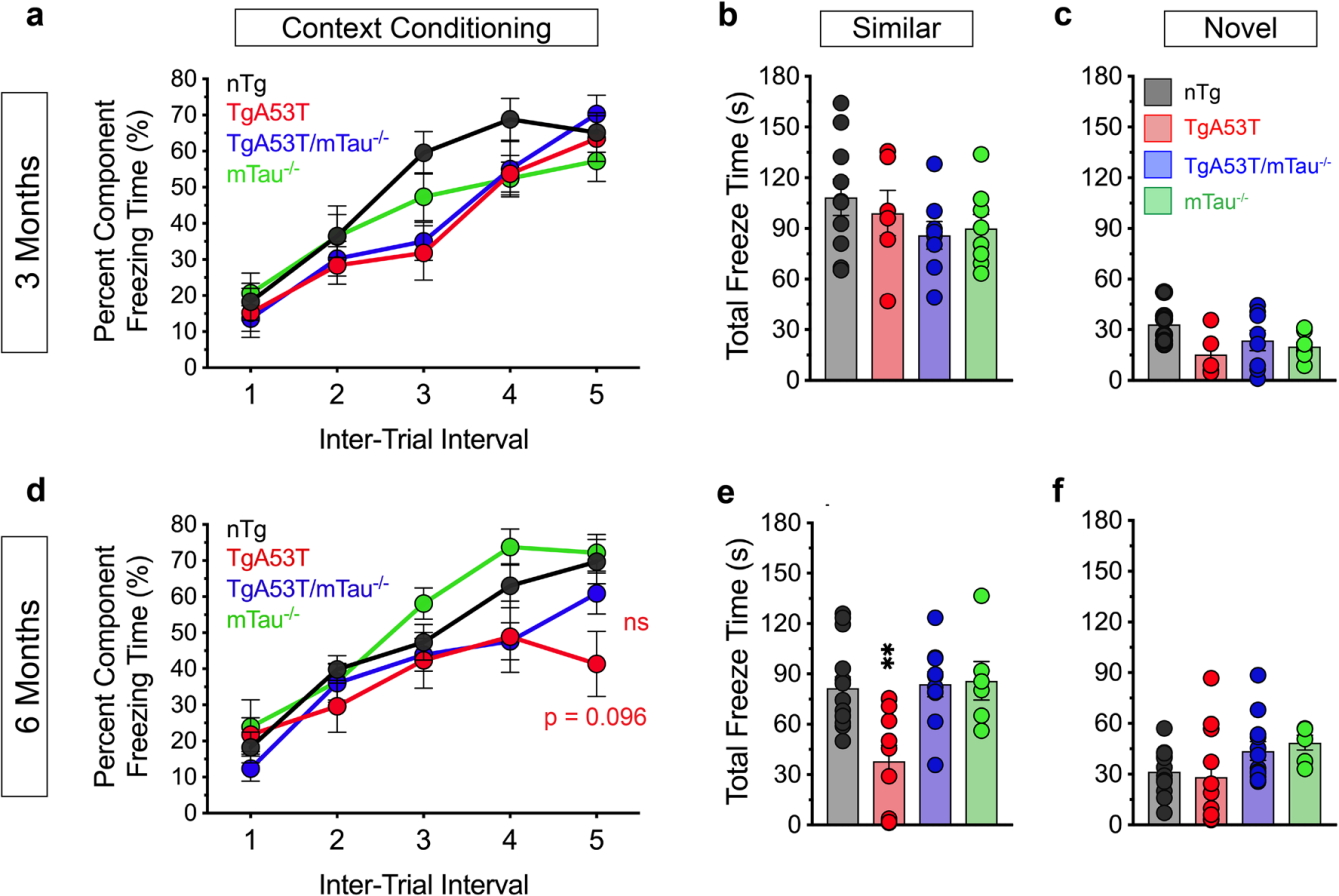
Mutant synuclein impairs hippocampal contributions to contextual fear conditioning in both age and tau-dependent manners. Contextual fear conditioning (CFC) in mice at 3 months (3M) (**a–c**) and 6 months (6M) of age (**d–f**). 3M conditioning trial (**a**): three-way repeated measures ANOVA with Geisser–Greenhouse correction and Tukey’s posthoc analysis revealed a significant effect of inter-trial interval [*F*_(3.200,89.59)_ = 67.91, *p* < 0.0001], no significant effect of hαS^A53T^ genotype [*F*_(1,28)_ = 2.123, *p* = 0.1562], no significant effect of mTau^−/−^ genotype [*F*_(1,28)_ = 0.2536, *p* = 0.6185], and no significant inter-trial interval*hαS^A53T^*mTau^−/−^ interaction [*F*_(4,112)_ = 1.075, *p* = 0.3723]. 6M conditioning trial (**d**): three-way repeated measures ANOVA with Geisser–Greenhouse correction and Tukey’s posthoc analysis revealed a significant effect of inter-trial interval [*F*_(3.175,120.6)_ = 52.72, *p* < 0.0001], a significant effect of hαS^A53T^ genotype [*F*_(1,38)_ = 5.429, *p* = 0.0252], no significant effect of mTau^−/−^ genotype [*F*_(1,38)_ = 0.7273, *p* = 0.3991], and no significant inter-trial interval*hαS^A53T^*mTau^−/−^ interaction [*F*_(4,152)_ = 2.257, *p* = 0.0655]. Percent of time spent freezing during conditioning trials (**a**, **d**) show similar responses in all genotypes. When mice are exposed to the similar environment, all genotypes show similar freezing responses at 3M (**b**), while 6M TgA53T mice freeze less than controls (**e**) [one-way ANOVA with Tukey’s posthoc analysis: *F*_(3,37)_ = 7.538, *p* = 0.0005], indicating defective CFC. In contrast, 6M TgA53T/mTau^−/−^ mice show normal memory (**e**). Responses to the novel environment are not different between groups at both 3M (**c**) and 6M (**f**). 3M: *n*_nTg_ = 8; *n*_TgA53T_ = 7; *n*_TgA53T/mTau_^−/−^ = 8; *n*_mTau_^−/−^ = 9. 6M: *n*_nTg_ = 13; *n*_TgA53T_ = 11; *n*_TgA53T/mTau_^−/−^ = 10; *n*_mTau_^−/−^ = 8. One- and three-way ANOVA: **p < 0.01. *ns* not significant. Error bars represent mean ± SEM

While TgA53T mice demonstrate intact spatial learning and memory relative to nTg littermates at 6 months of age, hippocampal LTP deficits occur by 6 months in this model [110]. Given that TgA53T mice may display mild BM learning deficits in early training days at 6 months of age, we hypothesized that TgA53T mice may exhibit more obvious tau-dependent deficits at earlier ages in other memory modalities involving hippocampal function. We first used contextual fear conditioning (CFC) to determine if TgA53T mice show deficits in this dual hippocampal and amygdala-dependent task [7, 76, 91]. 3-month-old TgA53T mice show normal learning and memory comparable to the nTg littermates (Fig. 2a–c). At 6 months of age, TgA53T mice respond similarly to foot shocks during the context conditioning trial as compared to nTg, TgA53T/mTau^−/−^, and mTau^−/−^ cohort counterparts (Fig. 2d), indicating that these mice do not exhibit gross sensorimotor abnormalities that could confound these findings. TgA53T mice exhibit significantly less freezing when exposed to the “similar” context as compared to the other three strains tested (Fig. 2e), indicating that CFC is impaired in 6-month-old TgA53T mice. All groups displayed reduced freezing tendencies in the “novel” setting, controlling for any sensorimotor differences (Fig. 2c, f). Significantly, TgA53T/mTau^−/−^ animals showed equivalent CFC capacities compared to control mice (nTg and mTau^−/−^), demonstrating that tau is required for mutant αS-dependent deficits in CFC.

Both the BM and CFC represent situations that subject mice to stressful environments to promote task performance. Thus, to reduce any confounds associated with the differential stress responses, we asked whether TgA53T mice have cognitive deficits in short-term memory using a spatial variant of the Y maze (YM), a less stressful assessment of spatial learning and memory [25]. Animals undergo an initial learning trial, where the “novel” arm is blocked off followed by a “recognition” trial, where all arms are open for exploration (Suppl. Figure 3a, Online Resources). The recognition trial takes advantage of an animal’s propensity to explore novel objects and environments and tests their capacity to discriminate between novel and familiar environments [6, 109]. As such, measuring the amount of time spent in the novel (N) versus familiar (F) arms of the YM provides an assessment of short-term spatial learning and memory. 6-month-old nTg mice spent significantly more time in the N arm, while littermateTgA53T mice spent equal amounts (chance) between N and F arms during the recognition trial (Suppl. Figure 3b, c, Online Resources). The hαS^A53T^-mediated reductions in spatial discrimination are suggestive of an impaired capacity for this hippocampal-dependent short-term spatial memory at 6 months of age. More importantly, the YM spatial discrimination deficit was reversed in TgA53T/mTau^−/−^ mice, indicating that endogenous tau expression is required for hαS^A53T^-associated impairments in both short- and long-term spatial learning and memory (Suppl. Figure 3b, Online Resources). In TgA53T mice, Lewy-like amyloid intraneuronal accumulations of hαS are found primarily the dorsal midbrain, cerebellar nuclei, brainstem, and spinal cord, accompanied by astrogliosis but no neurodegeneration [65]. However, these neuropathological changes are absent in cortical and limbic system structures, including the hippocampus and amygdala, of TgA53T mice brains [65] suggesting that hαS^A53T^-mediated neuronal dysfunction can occur in the absence of aggregate formation. This observation is consistent with another hαS^A53T^-expressing transgenic mouse line that develops motor and gait deficits but no αS-positive intracellular inclusions [38].

### Presymptomatic motor hyperactivity of TgA53T mice is tau-independent

**Fig. 3:**
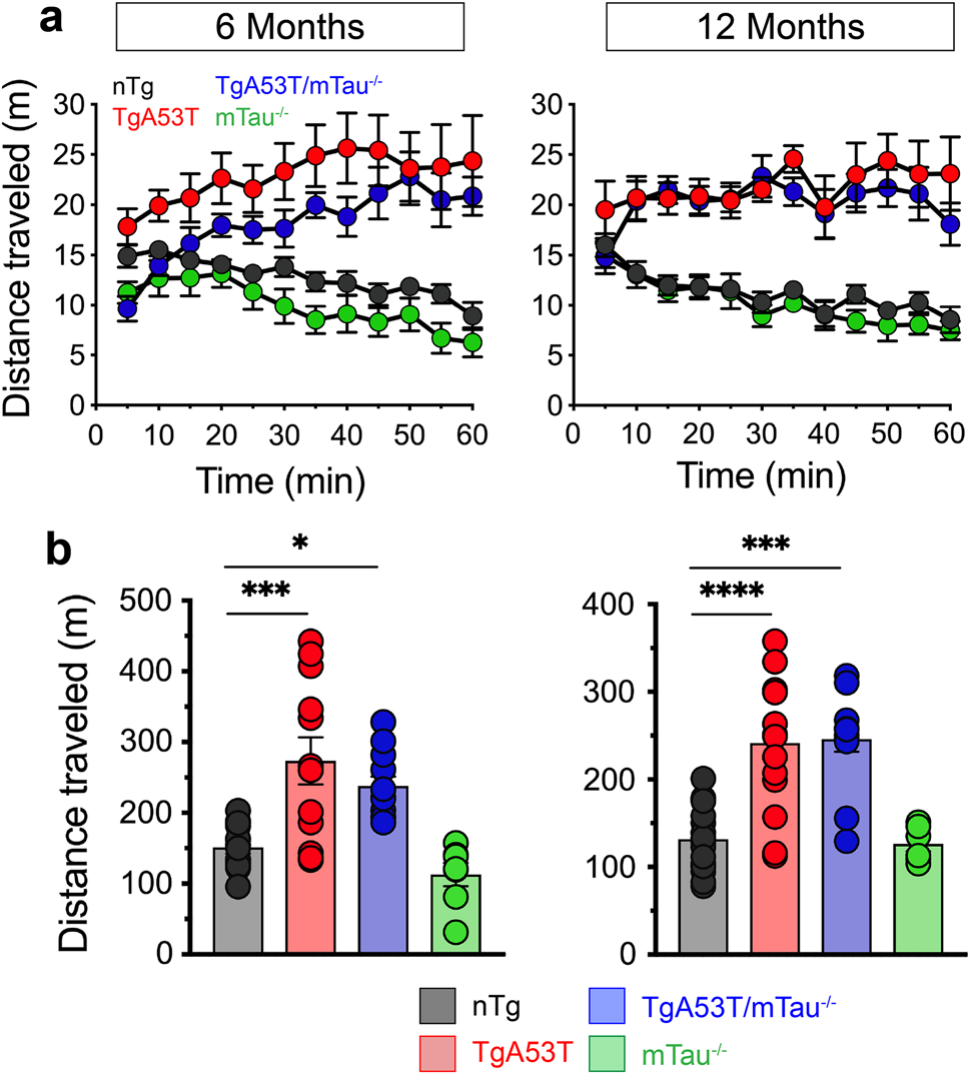
Locomotor hyperactivity in the TgA53T model is tau-independent. **a** Distance traveled (m), binned into 5-min intervals, for the entire 60-min activity trial. **b** Summary graph of total distance traveled during the entire 60-min trial. TgA53T and TgA53T/mTau^−/−^ mice exhibit increases activity compared to controls (nTg and mTau^−/−^). 6 months (6M): *F*_(3,40)_ = 11.47 (*p* < 0.0001), one-way ANOVA with Tukey’s posthoc analysis. 12 months (12M): *F*_(3,47)_ = 19.28 (*p* < 0.0001), one-way ANOVA with Tukey’s posthoc analysis. 6M: *n*_nTg_ = 11; *n*_TgA53T_ = 12; *n*_TgA53T/mTau_^−/−^ = 11; *n*_mTau_^−/−^ = 9. 12M: *n*_nTg_ = 13; *n*_TgA53T_ = 9; *n*_TgA53T/mTau_^−/−^ = 11; *n*_mTau_^−/−^ = 8. One-way ANOVA: *p < 0.05 and ***p < 0.001. Error bars represent mean ± SEM

Our results so far indicate that removing tau expression restores learning and memory deficits in TgA53T mice, particularly those associated with intact hippocampal function. However, TgA53T mice exhibit other presymptomatic abnormalities in advance of overt α-synucleinopathy-associated motor dysfunction presentation. In particular, TgA53T mice, but not TgWT and TgA30P, exhibit spontaneous locomotor hyperactivity associated with increased striatal D1 dopamine receptor sensitivity [41, 116, 119]. Thus, we tested whether endogenous tau was required for hαS^A53T^-dependent hyperactivity. As expected, TgA53T mice exhibit a progressive increase in locomotor hyperactivity as measured by distance traveled in the open-field arena at 6 and 12 months. This hyperactivity was tau-independent, as TgA53T and TgA53T/mTau^−/−^ mice exhibit similar levels of hyperactivity (Fig. 3a, b). While tau is required for mutant αS-mediated deficits in hippocampal function, we conclude that mutant αS-driven abnormalities associated with nigrostriatal circuitry appear tau-independent, suggesting a cell-type specificity of pathologic interactions between αS and tau.

### Tau is required for A53T αS-linked impairments in excitatory and evoked neuronal activity

**Fig. 4:**
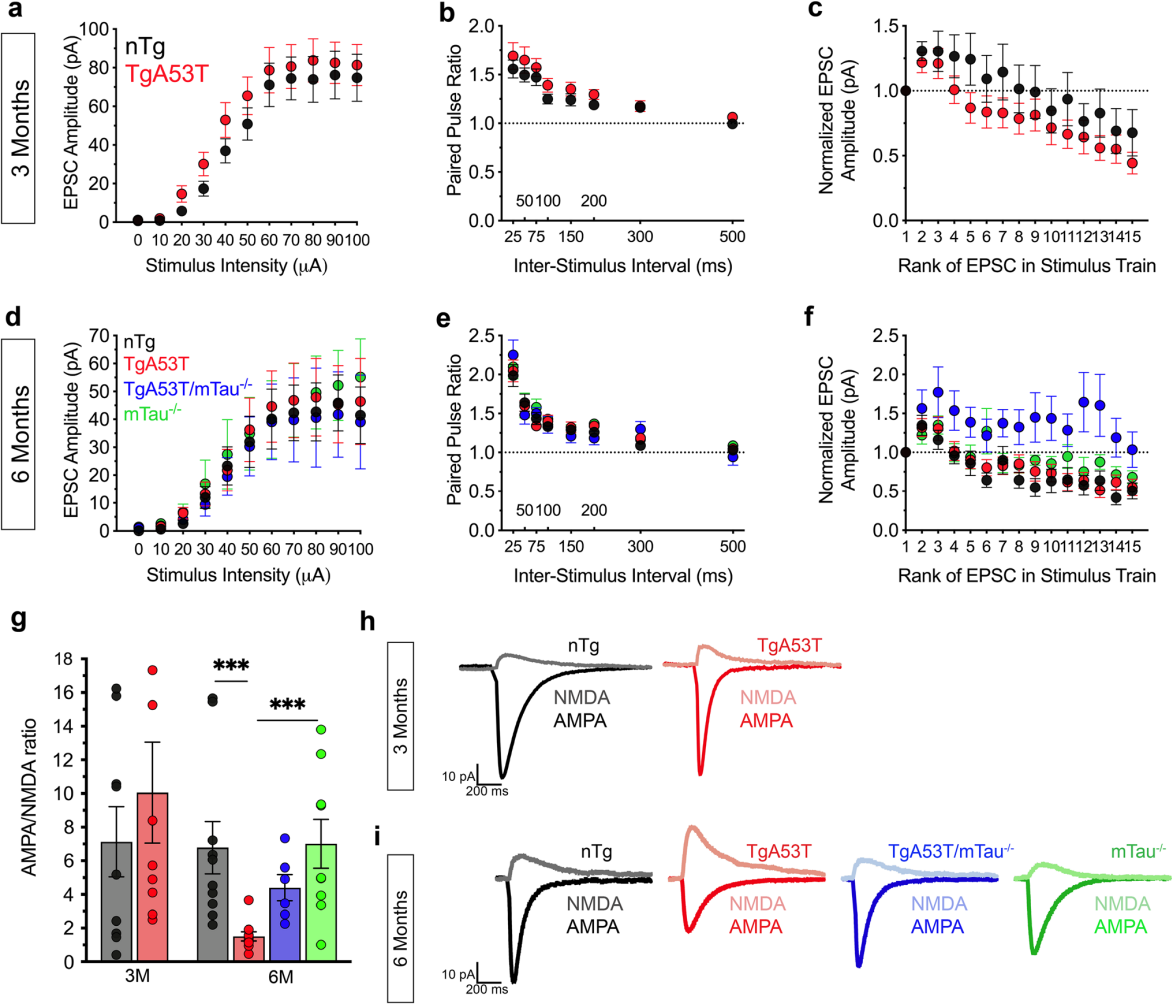
Tau is required for progressive loss of AMPAR-mediated neurotransmission in TgA53T neurons. Core excitatory postsynaptic current (EPSC) synaptic parameters assed in acute hippocampal slices from animals at 2–3 (3 months, 3M) and 5–6 (6 months, 6M) months of age, respectively: **a**, **d** Input–output curve. **b**, **e** Short-term potentiation measured via paired-pulse facilitation. **c**, **f** Short-term depression analyzed through synaptic fatigue. 3M: input–output: genotype *F*_(1,262)_ = 4.790, *p* = 0.0295, stimulus intensity *F*_(10,262)_ = 28.19, *p* < 0.0001, genotype*stimulus intensity interaction *F*_(10,262)_ = 0.1596, *p* = 0.9985; paired pulse: genotype *F*_(1,176)_ = 7.422, *p* = 0.0071; inter-stimulus interval *F*_(7,176)_ = 15.34, *p* < 0.0001; genotype*inter-stimulus interval interaction *F*_(7,176)_ = 0.1785, *p* = 0.9894; synaptic fatigue: genotype *F*_(1,360)_ = 14.26, *p* = 0.0002, EPSC in train *F*_(14,360)_ = 4.611, *p* < 0.0001, genotype*EPSC in train interaction *F*_(14,360)_ = 0.2468, *p* = 0.9978; all two-way ANOVA with Sidak’s posthoc analysis. 6M: Input–output: genotype *F*_(3,407)_ = 0.7457, *p* = 0.5253, stimulus intensity *F*_(10,407)_ = 13.90, *p* < 0.0001, genotype*stimulus intensity interaction *F*_(30,407)_ = 0.05744, *p* > 0.9999; paired pulse: genotype *F*_(3,318)_ = 0.8501, *p* = 0.4674, inter-stimulus interval *F*_(7,318)_ = 51.83, *p* < 0.0001, genotype*inter-stimulus interval interaction *F*_(7,318)_ = 0.7178, *p* = 0.8147; synaptic fatigue: genotype *F*_(3,645)_ = 42.33, *p* < 0.0001, EPSC in train *F*_(14,645)_ = 5.048, *p* < 0.0001, genotype*EPSC in train interaction *F*_(42,645)_ = 0.7835, *p* = 0.8360; all two-way ANOVA with Tukey’s posthoc analysis. 3M (mice/slices/cells): *n*_nTg_ = 3/7/13; *n*_TgA53T_ = 3/7/13. 6M (mice/slices/cells): *n*_nTg_ = 5/10/11; *n*_TgA53T_ = 5/9/11; *n*_TgA53T/mTau_^−/−^ = 4/8/8; *n*_mTau_^−/−^ = 4/11/11. Except for modest reductions in synaptic fatigue associated with the mTau^−/−^ genotype (**f**), there are no obvious differences in evoked synaptic parameters. **g** Ratio of amplitude of AMPA:NMDA currents in CA1 pyramidal neurons from nTg and TgA53T mice at 3M, and nTg, TgA53T, TgA53T/mTau^−/−^, and mTau^−/−^ at 6M. AMPA/NMDA: *F*_(3,31)_ = 5.044, *p* = 0.0058, by one-way ANOVA with Tukey’s posthoc analysis. 3M (mice/slices/cells): *n*_nTg_ = 3/6/9 cells; *n*_TgA53T_ = 3/6/9 cells. 6M (mice/slices/cells): *n*_nTg_ = 7/9/10 cells; *n*_TgA53T_ = 4/10/10 cells; *n*_TgA53T/mTau_^−/−^ = 3/7/7 cells; *n*_mTau_^−/−^ = 3/8/9 cells. Example AMPA and NMDA current traces from 3M (**h**) and 6M groups (**i**). While the AMPA/NMDA ratio is normal in 3-month-old TgA53T neurons, there is significant reduction in 6-month-old neurons. *t* test, and one- and two-way ANOVA: *p < 0.05 and ***p < 0.001. Error bars represent mean ± SEM

In our model for how mutant αS produces cognitive deficits, we propose that mutant αS causes tau-dependent postsynaptic deficits characterized by the loss of AMPAR and subsequent deficits in hippocampal glutamatergic signaling, LTP, and memory impairments [110]. However, it is unknown if these pathways are directly interrelated. We, therefore, examined if endogenous tau expression was also required for αS-mediated synaptic deficits using acute hippocampal slice recordings [110]. Furthermore, to determine if these neurophysiological deficits in TgA53T neurons are progressive, we examined hippocampal slices from mice at 2–3 months of age, where animals have intact cognition, and 5–6 months of age, where mice first display impairments in CFC and YM. Consistent with our previous study, neurons from all mice show similar profiles in analysis of basal synaptic transmission at both age points, including analysis of input/output curves and paired-pulse facilitation (Fig. 4a–f). Analysis of synaptic fatigue shows that neurons lacking tau, either mTau^−/−^ or TgA53T/mTau^−/−^, exhibit variable attenuation of stimulus-dependent reductions in EPSC (excitatory postsynaptic current) amplitude, suggesting that tau may be involved in the regulation of high-frequency synaptic transmission under physiological conditions (Fig. 4f). Neurons from 6-month-old TgA53T mice exhibit reduced synaptic strength as measured by comparing AMPAR currents to NMDA receptor (NMDAR) currents (AMPA/NMDA ratio) (Fig. 4g, h). By contrast, neurons from 2 to 3-month-old TgA53T mice show a normal AMPA/NMDA ratio when compared to nTg littermate controls (Fig. 4g, h), indicating that hαS^A53T^-mediated reductions in synaptic strength are progressive. Significantly, loss of tau expression in TgA53T/mTau^−/−^ reversed TgA53T-associated reductions in the AMPA/NMDA ratio (Fig. 4g–i) providing the first ex vivo confirmation that tau mediates mutant αS-driven deficits in synaptic function and that these deficits coincide with onset of cognitive dysfunction.

**Fig. 5:**
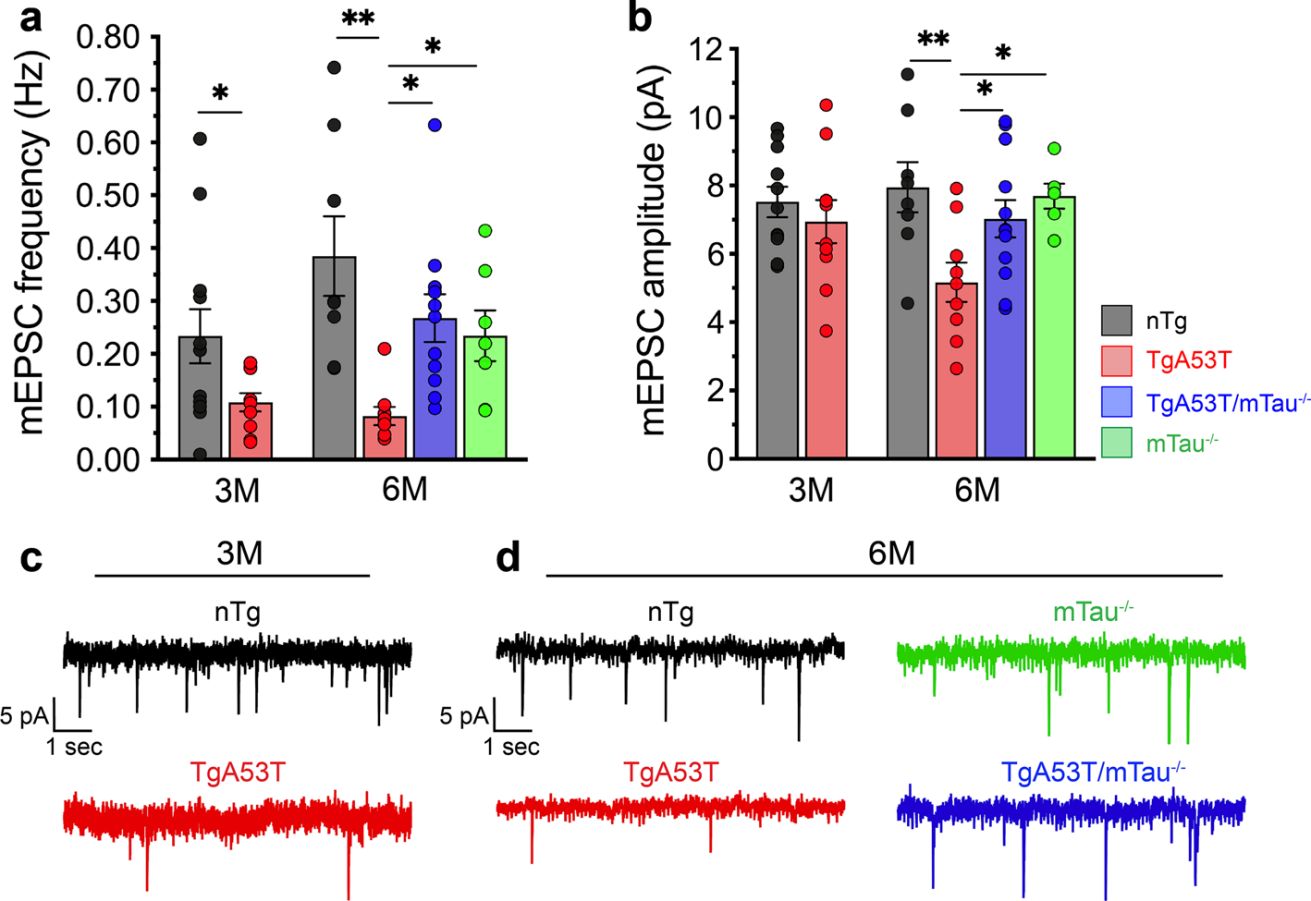
Spontaneous synaptic activity deficits in TgA53T neurons are reversed by loss of mTau expression. Spontaneous recordings of mini excitatory postsynaptic currents (mEPSCs) from CA1 pyramidal neurons in acute hippocampal slices were recorded from and analyzed for frequency (**a**) and amplitude (**b**) of mEPSCs from mice at 2–3 months (3M) and 5–6 months (6M) of age. 3M mEPSC frequency: *t* = 2.32, *df* = 13.51, *p* = 0.0364, by unpaired *t* test with Welch’s correction. 6M mEPSC frequency: *F*_(3, 31)_ = 6.213, *p* = 0.0020, by one-way ANOVA and Tukey’s posthoc analysis. 6M mEPSC amplitude: *F*_(3, 31)_ = 4.187, *p* = 0.0134, by one-way ANOVA and Tukey’s posthoc analysis. **c** Example mEPSC traces from 2 to 3-month-old TgA53T and nTg littermate controls. 3M (mice/slices/cells): *n*_nTg_ = 3/7/11; *n*_TgA53T_ = 3/6/10. 6M (mice/slices/cells): *n*_nTg_ = 5/8/8 cells; *n*_TgA53T_ = 3/7/9 cells; *n*_TgA53T/mTau_^−/−^ = 5/11/12 cells; *n*_mTau_^−/−^ = 3/6/7 cells. **d** Example mEPSC traces from 5 to 6-month-old nTg, TgA53T, TgA53T/mTau^−/−^, and mTau^−/−^ mice. The results show that reductions in mEPSC frequency in TgA53T neurons is not progressive from 3M to 6M, while reductions in mEPSC amplitude in TgA53T neurons is age-progressive over this time frame. *t* test and one-way ANOVA: *p < 0.05 and **p < 0.01. Error bars represent mean ± SEM

Overexpression of synuclein-family proteins including hαS^WT^, hαS^A53T^, and β-synuclein (βS) has been shown to reduce presynaptic vesicle release and neurotransmission [81]. However, hαS^A53T^ causes reductions in both spontaneous pre- and postsynaptic activities without detectable synapse loss [110]. Thus, we examined if tau expression modulates hαS^A53T^ reductions in synaptic activity by analyzing spontaneous neurotransmitter release and synaptic activity via recording of mini excitatory postsynaptic currents (mEPSCs). mEPSC frequency, reflecting the probability of neurotransmitter release from presynaptic vesicles, is reduced in TgA53T neurons at both 2–3 months and 5–6 months of age, confirming that inhibition of neurotransmission is a direct, primary synaptic effect of αS expression (Fig. 5a, c, d). More importantly, mEPSC amplitude, reflecting postsynaptic AMPAR function, was comparable between TgA53T and nTg neurons at 2–3 months of age, but shows significant reductions in slices from 5- to 6-month-old animals (Fig. 5b–d). These results, similar to memory deficits, indicate that postsynaptic deficits in TgA53T neurons are progressive with aging. Both mEPSC amplitude and frequency measured in neurons from TgA53T/mTau^−/−^ slices are not different from nTg and mTau^−/−^ controls, demonstrating that hαS^A53T^-mediated pre and postsynaptic deficits require tau expression. The fact that mTau^−/−^ neurons were not different from nTg neurons in these measures demonstrates that the reversal of mutant αS-dependent effects in TgA53T/mTau^−/−^ neurons is not due to simple additive effects.

It is possible that the findings in acute hippocampal slices could involve tau-dependent alterations in circuit development or compensation. To account for this possibility, we used dissociated hippocampal neuronal cultures to test whether loss of tau can reverse the synaptic changes observed in TgA53T neurons [110]. Neurophysiological analysis of primary cultures from TgA53T and TgA53T/mTau^−/−^ shows that loss of tau expression blocks the effects of mutant αS overexpression (Suppl. Figure 4, Online Resources), implicating tau as a direct mediator of mutant αS-induced pre- and postsynaptic changes.

**Fig. 6:**
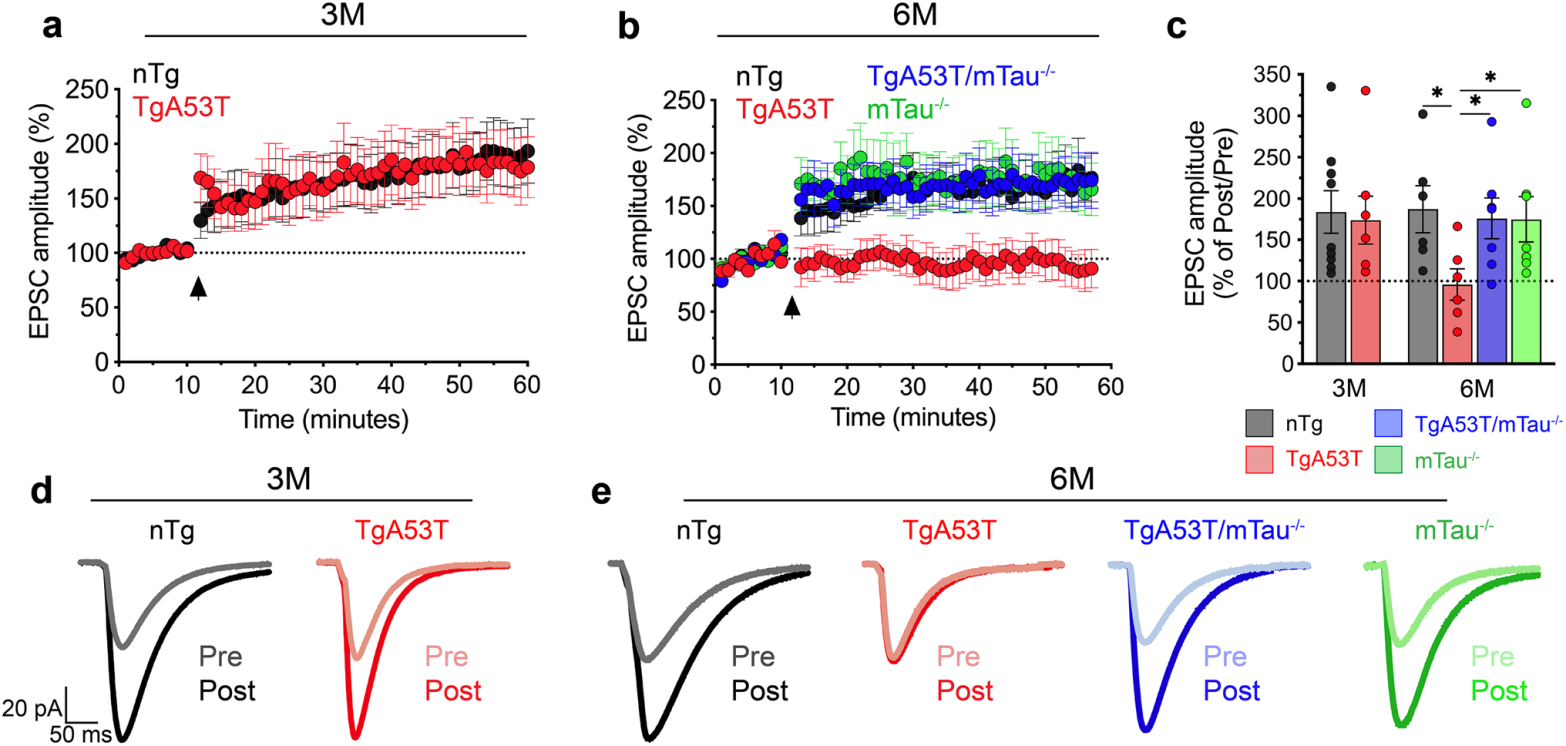
Progressive deficits in long-term potentiation in TgA53T neurons are tau-dependent and correlate with onset of cognitive impairments. Excitatory postsynaptic currents (EPSC) recorded via whole-cell recordings from hippocampal CA1 pyramidal neurons during long-term potentiation induced by high-frequency stimulation (HFS) of Schaffer collaterals of animals at 2–3 months (3M) (**a**) and 5–6 months (6M) of age (**b**). Arrowhead indicates application of HFS. **c** EPSC amplitudes 45 min following HFS (post), relative to baseline established prior to HFS (pre), from both 3M to 6M animals. 6M LTP EPSC: *F*_(3,22)_ = 2.584, *p* = 0.0262, by one-way ANOVA and Tukey’s posthoc analysis. 3M (mice/slices/cells): *n*_nTg_ = 4/9/9 cells; *n*_TgA53T_ = 4/7/7 cells. 6M (mice/slices/cells): *n*_nTg_ = 5/6/6 cells; *n*_TgA53T_ = 5/6/6 cells; *n*_TgA53T/mTau_^−/−^ = 3/7/7 cells; *n*_mTau_^−/−^ = 3/7/7 cells. These results show that TgA53T neurons exhibit normal LTP at 3M but severe LTP deficits at 6M. Furthermore, TgA53T/mTau^−/−^ neurons exhibit normal LTP at 6M. **d**, **e** Example EPSC traces from LTP experiments. Presented here are pre- and post-HFS in 3M neurons (**d**) and 6M neurons (**e**). *t* test and one-way ANOVA: *p < 0.05. Error bars represent mean ± SEM

Having established tau as a mediator of hαS^A53T^-driven postsynaptic deficits in spontaneous neurotransmission, we next asked if tau contributes to A53T αS-mediated deficits in long-term plasticity by evaluating hippocampal LTP induction [71]. Consistent with intact spatial learning and memory (Fig. 2; Suppl. Figure 2, Online Resources), LTP in TgA53T mice at 2–3 months is normal when compared to age-matched nTg littermates (Fig. 6a, c, d). At 6 months, cognitively impaired TgA53T mice display tau-dependent memory deficits (Fig. 2; Suppl. Figure 3, Online Resources) and LTP deficits (Fig. 6b, c, e). Taken together, our findings thus far demonstrate that tau is required for TgA53T-associated progressive impairments in synaptic activity and plasticity and spatial learning and memory. Moreover, our results show that presynaptic effects due to αS overexpression are not sufficient to cause memory deficits, suggesting that altered postsynaptic glutamatergic signaling is required for cognitive dysfunction in the TgA53T model.

### Tau does not alter expression or oligomerization of αS in forebrains of TgA53T mice

**Fig. 7:**
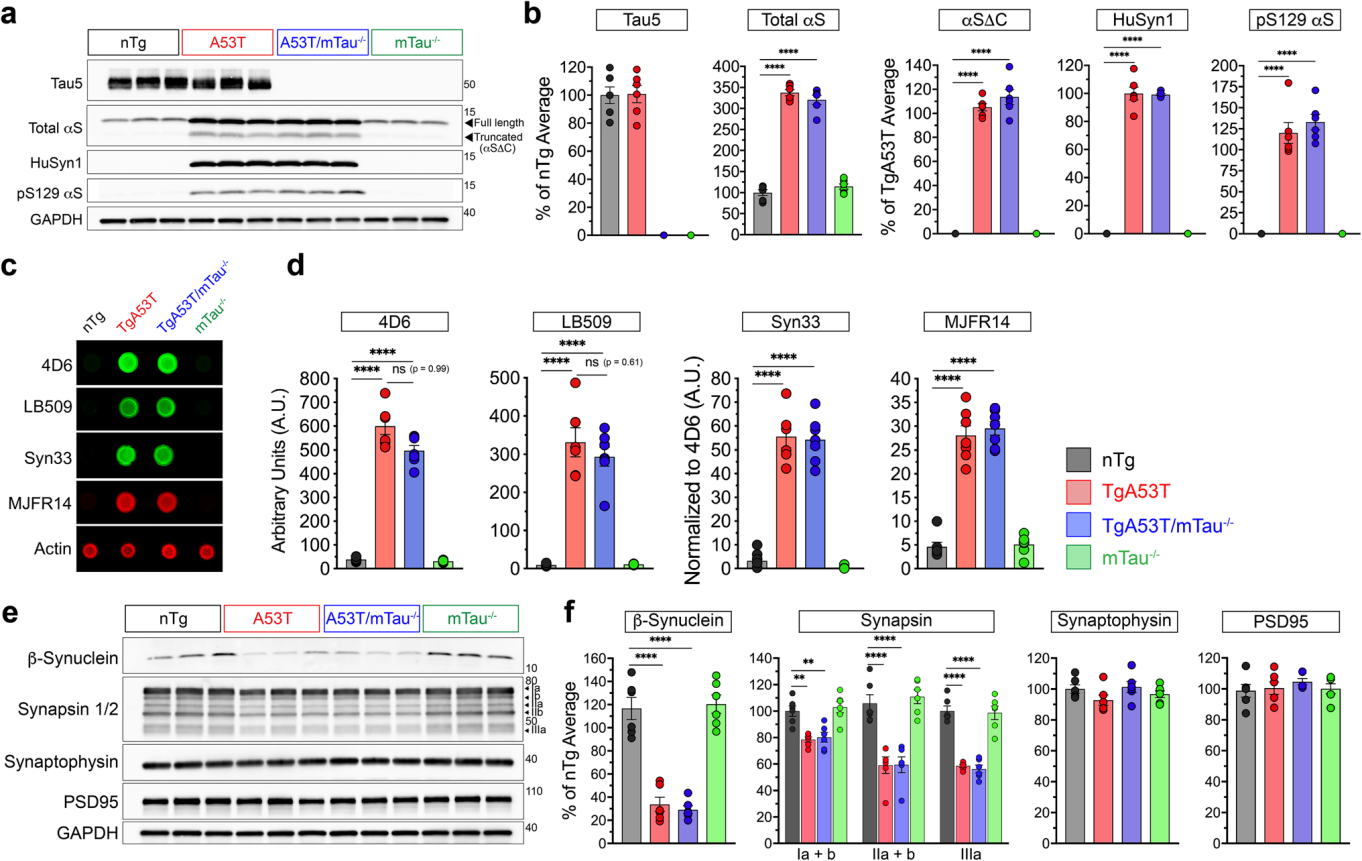
Tau-dependent synaptic and cognitive deficits in TgA53T mice are independent of expression or aggregate-specific changes in αS or key presynaptic proteins. **a** Representative western blot analysis of hippocampal lysates from 12-month-old mice. **b** Densitometry of hippocampal protein expression. For tau and total αS (full length), values were normalized to the average values for nTg samples within each gel. For truncated αS (αSΔC), human αS (HuSyn1), αS phosphorylated at Ser129 (pS129 αS), values were normalized to the average densitometric values of TgA53T samples within each gel. For western blot densitometry: one-way ANOVA with Tukey’s posthoc analysis. Total αS: *F*_(3,20)_ = 252.4, *p* < 0.0001. αSΔC: *F*_(3,20)_ = 335.9, *p* < 0.0001. HuSyn1: *F*_(3,20)_ = 616.2, *p* < 0.0001. pS129 αS: *F*_(3,20)_ = 88.70, *p* < 0.0001. While αS-associated protein levels are increased in TgA53T mice, the levels are not altered in TgA53T/mTau^−/−^ mice. *N* = 6 animals/genotype. **c** Representative dot blots from non-denatured 12-month-old hippocampal lysates for the epitopes associated with total αS (4D6), human αS (LB509), and various pathological αS oligomers (Syn33, MJFR14). Dot blots for additional αS oligomers and pathological tau are shown in Suppl. Figure 5 (Online Resources). **d** Dot blot densitometry for levels of total αS (4D6) and human αS (LB509), normalized to actin levels. For higher order αS species, Syn33 and MJFR14, densitometry values were normalized to the average densitometric values of TgA53T samples within each gel. For all dot blot densitometry: one-way ANOVA with Tukey’s posthoc analysis. 4D6: *F*_(3,23)_ = 232.9, *p* < 0.0001. LB509: *F*_(3,23)_ = 68.53, *p* < 0.0001. Syn33: *F*_(3,23)_ = 116.4, *p* < 0.0001. MJFR14: *F*_(3,23)_ = 101.1, *p* < 0.0001. *N* = 8 animals/genotype. While αS and αS oligomer species levels are increased in TgA53T mice, they are unchanged by tau removal in TgA53T/mTau^−/−^ mice. **e** Representative western blot images of presynaptic and postsynaptic proteins of interest in 12-month-old hippocampi. **f** Densitometry of hippocampal protein expression. Values were normalized to the average values for nTg samples within each gel. For western blot densitometry: one-way ANOVA with Tukey’s posthoc analysis. β-Synuclein: *F*_(3,20)_ = 49.86, *p* < 0.0001. Synapsin Ia + b: *F*_(3,20)_ = 8.501, *p* = 0.0008. Synapsin IIa + b. *F*_(3,20)_ = 9.482, *p* = 0.0004. Synapsin IIIa: *F*_(3,20)_ = 46.62, *p* < 0.0001. N = 6 animals/genotype. Densitometry shows that while β-synuclein and synapsin isoforms are decreased in TgA53T mice, they are not altered by loss of tau expression. Furthermore, the levels of synaptophysin and PSD95 are comparable in all animals, indicating a lack of synaptic loss. For all, values were normalized to the average densitometric values of nTg samples within each gel. One-way ANOVA: **p < 0.01 and ****p < 0.0001. *ns* not significant. Error bars represent mean ± SEM

Post-translational modifications of αS are believed to be integral to PD pathophysiology. Levels of aggregate-promoting C-terminally truncated αS (αSΔC) are increased by familial PD-linked A30P and A53T missense mutations [66]. Furthermore, phosphorylated αS at Serine 129 (pSer129) is an accepted marker of αS pathology in situ [32, 96]. Thus, we asked if tau-dependent synaptic and memory deficits and cognitive decline in TgA53T mice were due to alterations in hippocampal and cortical αS expression, increased pathogenic αS modifications, αS oligomer accumulation, or abnormal tau accumulation. Immunoblot analysis demonstrates that total hippocampal and cortical tau levels were not different between nTg and TgA53T lysates, indicating that alterations in tau expression are not responsible for tau-dependent deficits in TgA53T mice and neurons. Conversely, the loss of tau also did not alter total αS levels in nTg or TgA53T mice (Fig. 7a, b; Suppl. Figure 5a, c, Online Resources). In addition, both αSΔC and pSer129αS levels were unaffected by tau expression (Fig. 7a, b). Finally, consistent with the previous studies [22, 65, 66], very little levels of detergent-insoluble αS are present in hippocampal lysates and levels of detergent-insoluble αS are not altered by tau expression (Suppl. Figure 6a, b, Online Resources).

Analysis of total tau expression via Tau5 antibody indicates that TgA53T cortices and hippocampi express comparable tau levels to that of nTg brains (Fig. 7a, b; Suppl. Figure 5a, c, Online Resources). To determine if the synaptic deficits observed in TgA53T neurons were associated with increased tau phosphorylation [110], expression of phosphorylated tau species as recognized by AT8, CP13, and PHF1 antibodies was determined via concurrent immunoblot analysis [56]. These results demonstrate that TgA53T brains exhibit modest, but significantly increased levels of AT8, CP13, and PHF1-positive phosphorylated tau (Suppl. Figure 6c, e, Online Resources). As expected, no immunoreactivity to tau-related epitopes is observed in mTau^−/−^ animals. These results are consistent with our hypothesis that hαS^A53T^ drives synaptic deficits by phosphorylation-dependent mislocalization of tau into dendritic spines [110].

αS and tau also have the capacity to induce each other’s aggregation and polymerization into fibrillar amyloid structures [36] and αS oligomers can induce tau misfolding and downstream generation of oligomers [17, 34]. Thus, we next investigated if the levels of soluble αS and/or tau oligomers could be correlated with synaptic and memory deficits in TgA53T mice. In all cortical and hippocampal lysates, no general amyloid structures (OC), soluble oligomeric species (A11), or tau oligomers (T22) [62] were present at detectable levels (Fig. 7c, d; Suppl. Figure 7, Online Resources). As expected, a panel of antibodies against conformational-specific and higher order assemblies of αS showed increased levels in TgA53T lysates. However, they did not reveal any changes as a function of tau expression: Syn33 (oligomers, dimers, and higher molecular weight aggregates), F8H7 (oligomers from 70 kDa and above), and MJFR-14-6-4-2 (soluble oligomeric and fibrillar structures) [62, 69, 84, 101]. While the mutant αS-selective effects on synaptic function and behavioral deficits suggest that pathological αS species are involved, our results indicate that the tau-dependent effects are not due to simple modulation of αS expression and αS oligomerization. Collectively, based on our results, we propose that the effect of tau may be occurring downstream or independent of the pathological conversion of αS.

### Memory deficits in TgA53T mice occurs in the absence of synaptic degeneration

In vertebrates, αS is a part of the larger synuclein gene family that include homologs β- and γ-synuclein [33, 64, 74]. β-Synuclein (βS) can antagonize αS aggregation and toxicity [44] and ameliorating endogenous βS enhances pathological αS phosphorylation and aggregation [30]. Consistent with the previous findings [112], expression of αS is inversely correlated with βS accumulation in the brain (Fig. 7e, f; Suppl. Figure 5b, d, Online Resources). Interestingly, the reduction in cortical and hippocampal βS expression of TgA53T mice is not rescued by removal of endogenous tau (Fig. 7e, f; Suppl. Figure 5b, d, Online Resources).

Other αS transgenic mouse models that exhibit memory deficits are associated with various levels of neurodegenerative changes, including cortical αS pathology and cortical and hippocampal synaptic loss [45, 57]. While cortical and hippocampal pathology are not robust features of the TgA53T model, we examined whether the tau-dependent memory deficits in the TgA53T model are due to loss of synapses by evaluating the protein abundance of presynaptic (synaptophysin) and postsynaptic (PSD95) proteins. Biochemically, there were no differences in the amounts of either synaptic proteins across the four genotypes assessed at 12 months of age (Fig. 7e, f; Suppl. Figure 5b, d, Online Resources). We next extended our biochemical findings with immunostaining to probe for changes in hippocampal and synaptic structure. TgA53T hippocampi at 12 months show similar hippocampal structure as compared to age-matched nTg littermates via staining for neuronal nuclei (NeuN; Suppl. Figure 8a, Online Resources), demonstrating that overt neuronal loss is not required for hαS^A53T^-mediated cognitive decline. Furthermore, confocal images of somatodendritic (MAP2), presynaptic (synaptophysin), and postsynaptic (PSD95) structures qualitatively indicate intact synaptic structures in cognitively impaired 12 month TgA53T mice compared to nTg littermates (Suppl. Figure 8b, Online Resources). Collectively, our results show that neither neuronal loss nor synaptic degradation is responsible for the αS-mediated cognitive dysfunction in TgA53T mice.

In light of an apparent intact synaptic integrity synapses in TgA53T mice, increased human wild-type or mutant αS expression has been implicated in dysregulation of presynaptic function by selectively reducing levels of synapsins [61, 81]. Immunoblot analyses of cortical and hippocampal lysates confirm reduced levels of synapsin isoforms in TgA53T mice (Fig. 7e, f; Suppl. Figure 5b, d, Online Resources). However, suppressed synapsin levels were not restored by loss of tau expression in TgA53T/mTau^−/−^ brains, indicating that tau functions in a distinct pathway or downstream of synapsin expression (Fig. 7e, f; Suppl. Figure 5b, d, Online Resources). These results support our behavioral findings that αS-mediated presynaptic deficits alone are not sufficient for memory deficits.

### Synaptic and memory deficits in TgA53T mice associated with tau-dependent alterations in postsynaptic glutamate receptors

**Fig. 8:**
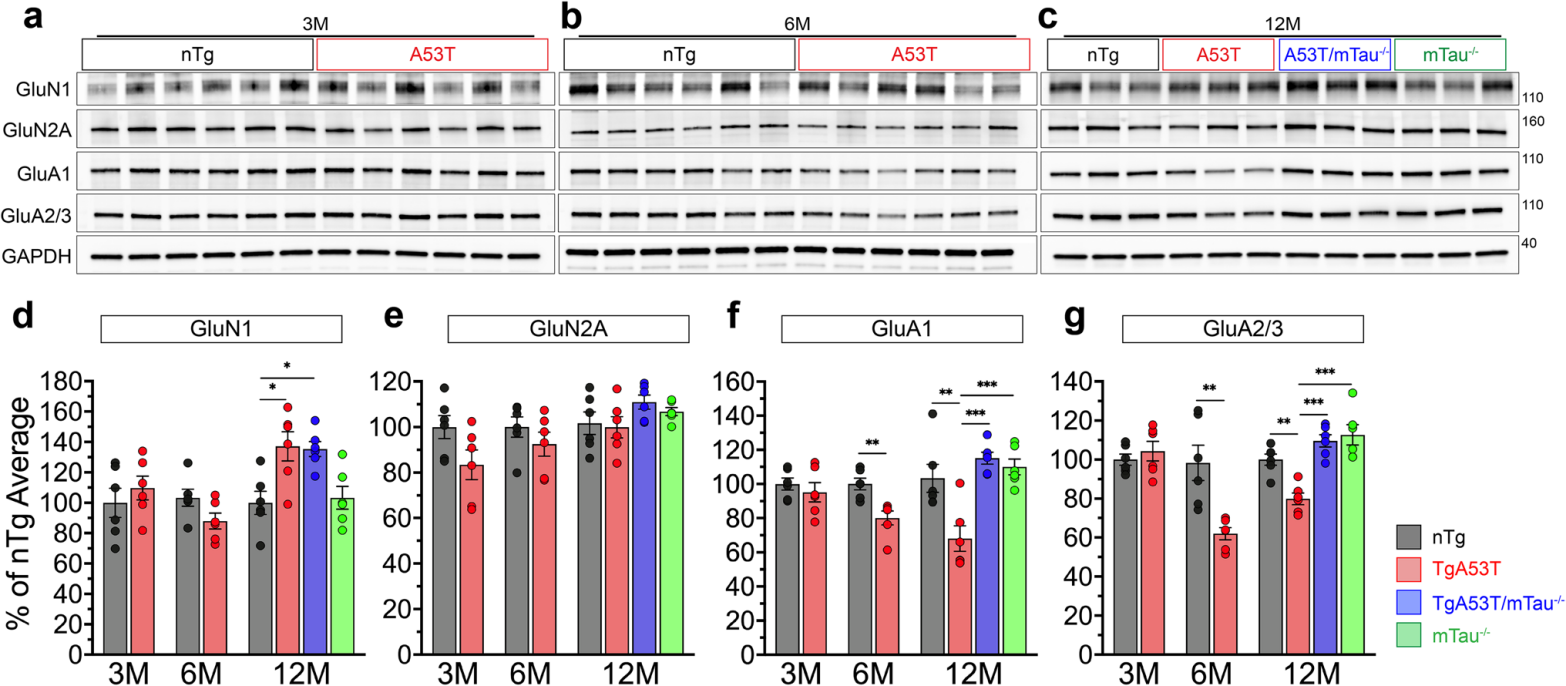
Progressive loss of AMPA receptor subunits in the TgA53T is tau-dependent. Representative western blot images of hippocampal lysates of AMPA (GluA) and NMDA (GluN) receptor subunits at 3 (**a**), 6 (**b**), and 12 (**c**) months of age (3M, 6M, and 12M, respectively). **d–g** Densitometry of immunoblots for AMPA and NMDA receptor subunits at 3M, 6M, and 12M. For all, values were normalized to the average densitometric values of nTg samples within each gel. For 3M and 6M: unpaired *t* test with Welch’s correction. For 12M densitometry: one-way ANOVA with Tukey’s posthoc analysis. 12M GluN1: *F*_(3,20)_ = 7.004, *p* = 0.0021. 6M GluA1: *t* = 3.773, *df* = 9.679, *p* = 0.0039. 12M GluA1: *F*_(3,20)_ = 11.55, *p* = 0.0001. 6M GluA2/3: *t* = 3.801, *df* = 6.180, *p* = 0.0085. 12M GluA2/3: *F*_(3,20)_ = 16.21, *p* < 0.0001. *N* = 6 animals/age/genotype. Compared to nTg mice, the NMDA receptor subunits are not decreased in TgA53T mice at all ages tested (**d**, **e**). However, AMPA receptor subunits are significantly decreased starting at 6M in TgA53T mice compared to nTg (**f**, **g**). Significantly, the loss of AMPA receptor subunits are reversed in TgA53T/mTau^−/−^ animals (**f**, **g**). *t* test and one-way ANOVA: *p < 0.05, **p < 0.01, and ***p < 0.001. Error bars represent mean ± SEM

The cellular and molecular mechanisms driving LTP are mediated by postsynaptic AMPAR and NMDAR [10, 71, 72]. Tau missorting to dendritic spines is associated with memory loss and impairments in postsynaptic AMPAR signaling in frontotemporal dementia and parkinsonism linked to chromosome 17 (FTDP-17), Alzheimer’s disease (AD) [47, 77]. We showed that hαS^A53T^-mediated tau missorting to dendritic spines is associated with calcineurin-dependent AMPAR internalization [110]. As the loss of hippocampal AMPARs likely contributes to deficits in LTP and memory, we sought to determine if reductions in AMPAR expression occurs in TgA53T mice. Immunoblot analysis displayed progressive reductions specific to hippocampal, but not cortical, AMPAR subunits (GluA1 and GluA2/3). Specifically, while AMPAR levels are not different between 3-month-old nTg and TgA53T mice, they are significantly reduced in TgA53T hippocampi at 6 and 12 months of age (Fig. 8a–c, f, g; Suppl. Figure 9, Online Resources). These hαS^A53T^-mediated deficits are tau-dependent as TgA53T/mTau^−/−^ hippocampi displayed levels of AMPAR expression similar to nTg and mTau^−/−^ controls at 12 months of age. Thus, loss of hippocampal AMPAR levels correlates with age- and tau-dependent onset of synaptic and cognitive deficits. To determine the selectivity of AMPAR changes, we also examined NMDAR levels, receptors essential for LTP but not functionally altered by tau mislocalization to spines [47]. GluN1 and GluN2A expression remained unchanged in cognitively intact 3-month-old or impaired 6-month-old TgA53T mice compared to nTg controls (Fig. 8a–e; Suppl. Figure 9, Online Resources). GluN1 expression was increased in 12-month-old TgA53T hippocampi and cortices, but, unlike AMPAR subunit expression changes, was neither changed by the status of tau expression (Fig. 8d). Collectively, these results parallel our neurophysiological findings and further our proposal that the TgA53T model is associated with the selective impairments in hippocampal postsynaptic AMPAR signaling in a tau-dependent manner, while NMDARs are largely unaffected or slightly increased in multiple brain regions.

AD-associated amyloid-β (Aβ) oligomers have been shown to alter postsynaptic signaling through binding to cellular prion protein (PrP^C^) that produces downstream Fyn activation and phosphorylation of the GluN2B subunit of NMDARs [63, 114]. It has recently been proposed that αS oligomers causes cognitive deficits through a mechanism involving activation of the cellular prion protein (PrP^C^), Fyn, and GluN2B [31]. We, therefore, examined whether PrP^C^-Fyn-GluN2B signaling is altered in the TgA53T model. In contrast to this recent study [31], TgA53T mice did not show increased PrP^C^ expression, an established mechanism for mediating downstream increases in Fyn and GluN2B phosphorylation and activation [31, 114, 115] (Suppl. Figure 10, Online Resources). We also determined that Fyn phosphorylation and activation was not increased in the TgA53T model (Suppl. Figure 11a, b, Online Resources). The final output for pathological PrP^C^ and Fyn signaling is increased NMDAR signaling through phosphorylation of GluN2B by Fyn. Consistent with the absence of increased PrP^C^ expression and Fyn activation, phosphorylation-inducing activation of GluN2B was not detected in TgA53T mice with either intact or impaired cognition (Suppl. Figure 11c, d, Online Resources). Taken together, our results do not support involvement of PrP^C^-Fyn-GluN2B signaling in the memory deficits observed in TgA53T mice. Our conclusion is also supported a recent study, showing that PrP^C^ neither binds nor mediates the toxic effects of αS oligomers [58].

### TgA53T mice exhibit progressive hippocampal inhibitory circuit remodeling

**Fig. 9:**
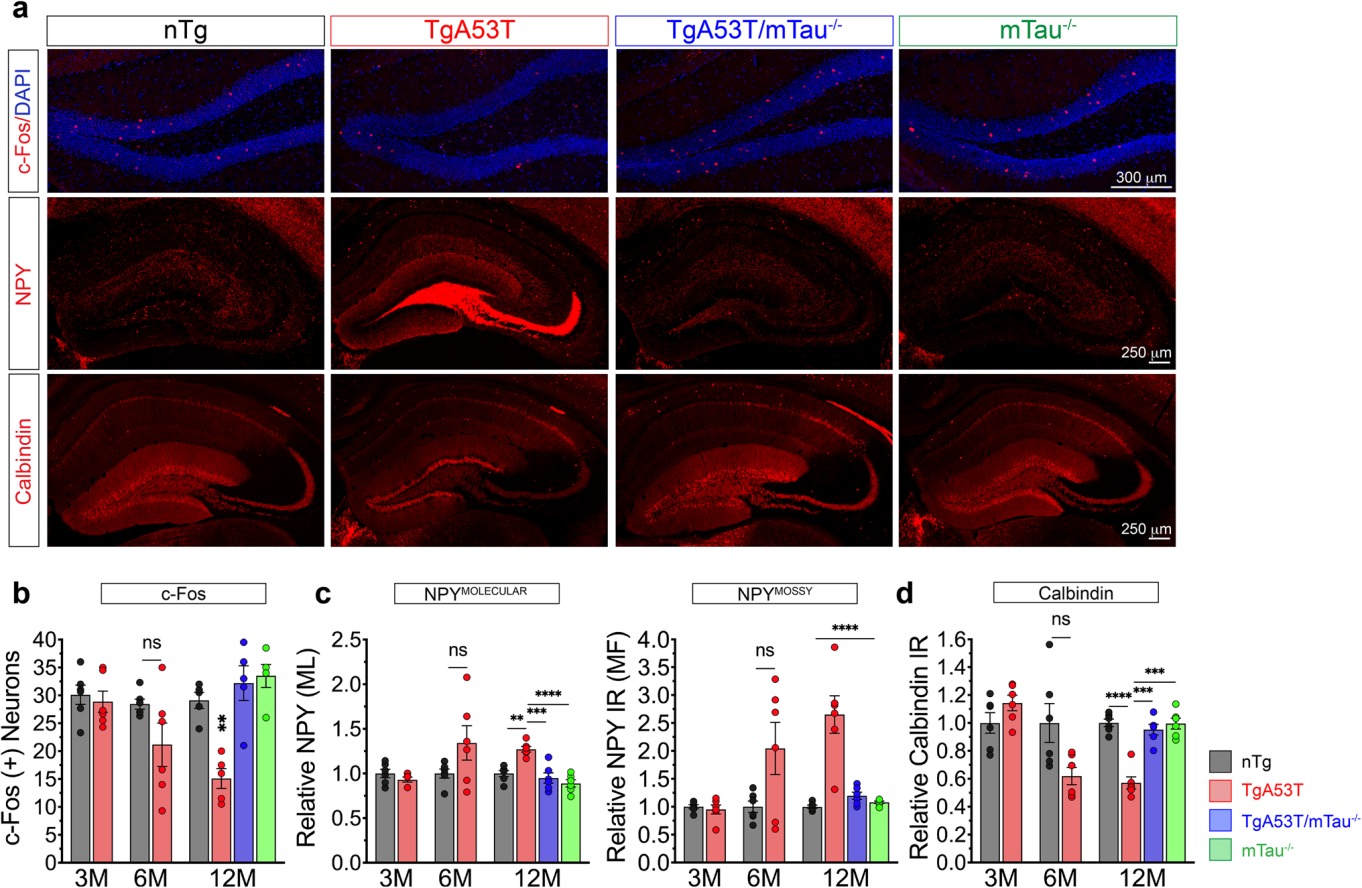
Progressive alterations in inhibitory circuits in the dentate gyrus of TgA53T mice is tau-dependent and correlate with the onset of synaptic and memory deficits. **a** Representative images from dentate gyri and hippocampi of 12-month-old (12M) nTg, TgA53T, TgA53T/mTau^−/−^, and mTau^−/−^ mice. Similar images for 3-month-old (3M) and 6-month-old (6M) are shown in Supplementary Fig. 11 (Suppl. Figure 11, Online Resources). c-Fos scale bar 300 μm. NPY and calbindin scale bar 250 μm. Quantification of immunoreactivity (IR) via cell counting (**b** c-Fos) or densitometry (**c** NPY in the Molecular Layer “Molecular”; NPY in the Mossy Fiber pathway, “Mossy”, and **d** calbindin) at 3M, 6M, and 12M. 6M: c-Fos: *U* = 7, *p* = 0.0844; NPY-molecular: *U* = 10, *p* = 0.2403; NPY-Mossy: *U* = 11, *p* = 0.3905; Calbindin: *U* = 6, *p* = 0.0649. 12M: c-Fos: *F*_(3,16)=_ 14.88, *p* < 0.0001; NPY-molecular: *F*_(3,16)_ = 15.97, *p* < 0.0001; NPY-Mossy: *F*_(3,16)_ = 19.77, *p* < 0.0001; Calbindin: *F*_(3,16)_ = 19.03, *p* < 0.0001. 3M analysis: unpaired *t* test with Welch’s correction. 6M analysis: Mann–Whitney *t* test. 12M analysis: one-way ANOVA with Tukey’s posthoc analysis. *N* = 6 animals/age/genotype, *n* = 8 sections/animal. In 3M TgA53T mice, the levels of synaptic activity markers are comparable to nTg mice. In 6M TgA53T mice, two distinct populations of mice exist: TgA53T and TgA53T^INT^ (analyzed in Suppl. Figure 11, Online Resources). All 12M TgA53T mice exhibit aberrant network remodeling which was reversed in TgA53T/mTau^−/−^ mice. *t* test and one-way ANOVA: *p < 0.05, **p < 0.01, ***p < 0.001, and ****p < 0.0001. *ns* not significant, *IR* immunoreactivity, *ML* molecular layer, *MF* mossy fiber. Error bars represent mean ± SEM

Disruption of neuronal networks due to chronic network hyperactivity has been hypothesized to be a potential mechanism contributing to cognitive decline in neurodegenerative diseases [87], particularly via increased spontaneous epileptiform activity and compensatory remodeling of networks [20, 88, 107]. Thus, we asked if TgA53T mice exhibit evidence of hippocampal circuit remodeling typical of chronic network hyperactivity [85, 88]: loss of c-Fos positive dentate granule cells, ectopic neuropeptide Y (NPY) expression in the mossy fiber pathway and molecular layer of dentate gyrus, and reduction in calbindin in the granule cells of dentate gyrus and stratum radiatum of CA1 [79, 88, 95]. At 3 months of age, cognitively intact TgA53T mice display c-Fos, NPY, and calbindin staining indistinguishable from nTg mice (Fig. 9c, e; Suppl. Figure 12a, f, Online Resources). These results indicate that when mice display normal postsynaptic function and memory, hippocampal circuits have not been exposed to chronic network hyperactivity. At 6 months, two distinct populations of TgA53T are observed: one displaying circuit remolding consistent with chronic network hyperactivity and another “intermediate” group without circuit remodeling (Fig. 9a, c, e; Suppl. Figure 12b–e, g, Online Resources). By 12 months of age, all TgA53T mice show prominent network changes indicative of chronic epileptic activity (Fig. 9b–d; Suppl. Figure 12 h, Online Resources). Importantly, these age-dependent, αS-mediated alterations in hippocampal circuits are absent in the 12-month-old TgA53T/mTau^−/−^ mice. These results indicate that hippocampal network remodeling in the TgA53T model starts between 3 and 6 months of age, likely as a compensatory response to network hyperactivity. The variability observed in 6-month-old TgA53T mice is consistent with animals undergoing a transition period. Furthermore, the TgA53T-linked inhibitory hippocampal circuit remodeling is absent in TgA53T/mTau^−/−^ animals, indicating that hαS^A53T^-associated network hyperactivity, along with progressive synaptic and memory deficits, requires endogenous tau expression.

## Discussion

Dementia associated with α-synucleinopathy, particularly PDD and DLB, is one of the leading sources of global disability from neurological disorders [28, 53]. Currently, there are no effective therapies for managing PDD and DLB. Thus, understanding the cellular and molecular mechanisms of dementia in PDD and DLB will facilitate the development of new treatments. Previously, we proposed a model, where hαS^A53T^ induces tau mislocalization to dendritic spines, leading to postsynaptic deficits in AMPAR signaling [110]. However, it was not known if these tau-related changes are directly causative for deficits in synaptic plasticity and learning and memory in the TgA53T mouse model. In our present study, we provide the first in vivo evaluation of the mechanistic relationships between αS abnormalities, tau expression, synaptic function, and memory. Our data show that the removal of endogenous tau expression reverses a range of synaptic and memory impairments in TgA53T mice. Furthermore, our results indicate that the effect of tau may be downstream or independent of potentially pathogenic αS oligomeric or fibrillar species. Because TgA53T-associated deficits in synaptic function and learning and memory are not connected with hippocampal neurodegeneration, it is likely that tau regulates hαS^A53T^ alterations in synaptic physiology by perturbing neuronal function. In particular, our results indicate that tau-dependent loss of AMPAR function is an early event that is tightly correlated with LTP deficits, evidence of hippocampal network remodeling suggestive of chronic hyperactivity, and cognitive dysfunction in the TgA53T model.

A convergence of αS, tau, and Aβ pathologies has been observed in PDD and DLB patients with increased cognitive impairment, suggesting a mechanism for neuronal dysfunction in α-synucleinopathies involving more than αS pathology [23, 51]. Significantly, dementia, cortical and hippocampal αS pathology, and tau pathology are often noted features of PD patients with A53T mutation in αS [11, 29, 39, 73, 106]. In this study, we expand on the interaction between αS and tau by showing that physiological tau expression independent contributes to neurophysiological and memory deficits in TgA53T mice. While we were not able to document accumulation of pathological tau oligomers, others have shown that pathological accumulation of tau oligomers in another TgA53T mouse model [34], suggesting that tau may act downstream of αS pathology. Our results also add to the role of endogenous tau expression as a mediator of neuropathological phenotypes in AD models [52, 94, 95, 118]. The requirement of tau for memory deficits and the immunohistochemical markers of chronic epileptiform activity in the TgA53T model is reminiscent of studies in the hAPP-J20 transgenic mouse model of AD, where loss of tau expression reversed memory deficits and epileptic activity [88, 94, 95]. In hAPP-J20 mice, chronic epileptiform activity is mechanistically linked to cognitive deficits. Thus, it is likely that network hyperactivity, as indicated by age-dependent remodeling of inhibitory networks in TgA53T mice, also contributes to memory deficits in the TgA53T mouse model. Significantly, another model of αS-dependent memory deficits (Thy1-hαS: line 61) [45] also shows increased epileptic activity, which was partially dependent on endogenous mouse tau expression [79]. However, it is unknown if tau is required for memory deficits in the Thy1-hαS model or in other models of αS-dependent synaptic and memory deficits. The pathologic link between epileptic activity and memory deficits may be relevant for understanding DLB as both AD and DLB are associated with higher incidences of seizures and signs of seizure-like network hyperactivity [9, 75, 78, 79, 88, 95, 107, 117].

The progressive cognitive decline in TgA53T mice first observed at 6 months of age appears to correlate primarily with the loss of AMPAR expression leading to deficits in postsynaptic activity and LTP. Interestingly, TgA53T mice at this age point do not consistently display the molecular signature for chronic network hyperactivity of reductions in dentate granule cell c-Fos expression, increased ectopic NPY expression, and calbindin depletion in dentate granule cells, suggesting that the synaptic and memory deficits in the TgA53T model seem to precede inhibitory network remodeling. However, because network remodeling is a likely response to chronic and aberrant network hyperactivity [85–87], we propose that the onset of this epileptiform activity is coincident with other synaptic deficits. It will be of significant future interest to determine the causal relationship between synaptic deficits, epileptic activity, and memory deficits, as it has been studied in the hAPP-J20 model [97].

While several transgenic Aβ and αS mouse models share a final common outcome of memory loss through impairments in synaptic and neural network function, the mechanistic details appear to differ. One hypothesis for Aβ-mediated cognitive decline involves postsynaptic dysfunction via activation of the PrP^C^–Fyn pathway, leading to phosphorylation and activation of the NMDAR subunit GluN2B, which, in turn, culminates in excitotoxicity and spine loss [37, 63, 95, 114]. This pathway has also been recently implicated in memory loss associated with α-synucleinopathies [31]. However, in our studies, we did not observe PrP^C^–Fyn–GluN2B activation in TgA53T mice, indicating that hαS^A53T^ produces progressive postsynaptic and cognitive deficits through a mechanism independent of this pathway. Interestingly, in the hAPP-J20 model of AD, which, like TgA53T model of α-synucleinopathy, requires tau as a mediator of synaptic dysfunction and memory loss [94], PrP^C^, and Fyn activation appears to be mechanistically disconnected as tau-dependent cognitive decline in hAPP-J20 mice is unaffected by PrP^C^ ablation [21]. Furthermore, this view is consistent with a recent report [58] showing that, in contrast to the findings of Ferreira et al. [30], PrP^C^ does not mediate the detrimental effects of αS oligomers. Our results suggest that synaptic and memory deficits in the TgA53T model appear to be independent of alterations in NMDAR but associated with decreased AMPAR subunit expression. Finally, consistent with the lack of Fyn–GluN2B activation, we do not observe a significant loss of postsynaptic structures in aged TgA53T mice.

Pathological αS can spread throughout the central nervous system, including cortical and hippocampal neurons, in a hierarchal progression that appears to follow functionally connected regions [12, 13], raising the possibility that certain neurodegenerative processes in PD may be produced by abnormal network activity. Supporting this mechanistic view, studies link PDD and DLB with LNs in the hippocampal CA2/3 region [4, 42]. Here, we provide evidence, where pathogenic αS causes reduced synaptic activity at specific excitatory synapses in the hippocampus, leading to deficits in AMPAR signaling and LTP. However, we also show that pathogenic hαS^A53T^ concurrently elicits aberrant excitatory activity as indicated by prominent compensatory remodeling of the inhibitory hippocampal circuits. One hypothesis to reconcile these seemingly contradictory observations is that depression of excitatory synaptic activity may be caused by a compensatory synaptic scaling mechanism reminiscent of the pro-excitatory effects of Aβ. For example, while acute oligomeric Aβ or αS application has been shown to transiently increase AMAPAR and NMDAR on the neuronal surface [31, 98], prolonged Aβ oligomer exposure decreases AMPAR and NMDAR and suppresses synaptic plasticity [49, 102].

In our study, deficits in postsynaptic function, LTP, and memory are selectively associated with the expression of hαS^A53T^. Since increased expression of α- or β-synucleins lead to presynaptic deficits [81, 110], we conclude that αS-associated presynaptic deficit is not sufficient to cause LTP and cognitive decline. However, memory deficits have been documented in other transgenic mouse models expressing hαS^WT^ or hαS^A30P^ under the Thy-1 promoter [45]. Thus, under appropriate circumstances, it is clear that multiple hαS variants can cause cognitive dysfunction. Currently, it is unknown if the postsynaptic deficits observed in our TgA53T animals are associated with memory deficits in these other TgWT or TgA30P mouse models. However, increased epileptic activity has been documented in the TgWT Thy1-hαS model (line 61) [79], suggesting possible common mechanisms underlying αS-dependent synaptic and network deficits. We propose that a number of factors, including variations in transgene expression, background of mouse strains, housing conditions, and testing conditions and parameters could all contribute to the accumulation of toxic αS species that cause of memory deficits in TgWT and TgA30P models. It will be of significance in the future to determine if tau is required for memory deficits in multiple transgenic mouse models of α-synucleinopathy.

Although synaptic loss is the major correlate for memory loss in AD patients [27, 99, 100, 111], clinical and pathological studies suggest that cognitive deficits in PDD/DLB occur in the absence of large-scale loss of hippocampal neurons [11, 46, 50, 55]. Furthermore, while the amount of tau pathology correlates with the severity of dementia [26], tau pathology per se is not as strongly linked to the incidence of dementia in PDD and DLB as αS or Aβ pathology [42, 50, 53, 54]. Significantly, the *MAPT* H1 haplotype increases risk for PDD and DLB [40, 83]. Since the *MAPT* H1 haplotype is thought to increase tau expression [80], it is possible that basal tau expression, independent of tau pathology, contributes to αS-dependent memory impairments in humans.

Prior to our current study, it was previously unknown how pathologic and genetic observations associated with PDD and DLB are related to the development of deficits in synaptic plasticity that underlies cognitive dysfunction. We provide evidence that the TgA53T model exhibits progressive, tau-dependent, memory loss associated with postsynaptic AMPAR dysfunction in the absence of overt synaptic loss and hippocampal neurodegeneration. More importantly, our results suggest that endogenous tau is required for the specific synaptic and memory deficits observed in TgA53T mice. In addition, we propose that hαS^A53T^ causes tau-dependent synaptic alterations that lead to increased network hyperactivity that contribute to and exacerbate memory deficits (Suppl. Figure 13, Online Resources). Our results, combined with the previous studies in AD models, suggest that network hyperexcitability represents a novel therapeutic target for treating this driver of cognitive dysfunction in α-synucleinopathies. Collectively, this study introduces a mechanism that identifies key proteins and mechanisms that could mediate the neuronal dysfunction underlying memory deficits in PDD and DLB. Ultimately, building a more complete picture of how pathologic αS produces memory deficits is essential for developing novel therapeutic strategies for PDD and DLB.

## Electronic supplementary material

Below is the link to the electronic supplementary material.

Supplementary material 1 (PDF 18968 kb)
