# Supplementary material for: Tau is required for progressive synaptic and memory deficits in a transgenic mouse model of α-synucleinopathy
Source: Acta Neuropathol. 2019 Jun 6;138(4):551–74. doi: 10.1007/s00401-019-02032-w (PMC6778173; doi:10.1007/s00401-019-02032-w)
Supplement: Supplementary file 1 — Supplementary material 1 (PDF 18968 kb) [file 401_2019_2032_MOESM1_ESM.pdf]

Tau is required for progressive synaptic and memory deficits in a transgenic mouse model of α-synucleinopathy

Balvindar Singh[1,2], Ana Covelo[3], Héctor Martell-Martínez[3], Carmen Nanclares[3], Mathew A. Sherman[3], Emmanuel Okematti[3], Joyce Meints[3], Peter J. Teravskis[3], Christopher Gallardo[4], Alena V. Savonenko[9], Michael A. Benneyworth[3,5,6], Sylvain E. Lesné[3,5,7], Dezhi Liao[3,5], Alfonso Araque[3,5], Michael K. Lee[3,5,8]

Affiliations

1. Medical Scientist Training Program, 2. Graduate Program in Neuroscience, 3. Department of Neuroscience; 4. Graduate Program in Pharmacology, 5. Institute for Translational Neuroscience, 6. Mouse Behavior Core, 7. N. Budd Grossman Center for Memory Research and Care, University of Minnesota, MN, USA; 8. Geriatric Research Education and Clinical Center, Minneapolis Veterans Affairs Health Care System, Minneapolis, MN, USA; 9. Department of Pathology, Johns Hopkins University, MD, USA

Loading Controls	Company	Reference	Use
Actin	Sigma-Aldrich	A2066	WB
GAPDH (GA1R)	Invitrogen	MA5-1738	WB
GAPDH (D16H11)	Cell Signaling	5174	WB
**α-Synuclein Species**	**Company**	**Reference**	**Use**
α-Synuclein (total)	BD Transduction	610787	WB
4D6 (total)	BioLegend	834304	DB
Phospho-serine 129 αS (pS129 αS)	Abcam	Ab51253	WB
F8H7 (conformation-specific oligomers)	Dr. Rakez Kayed (gift)	Sengupta et al., 2015	DB
FILA-1 (fibrillar αS)	Dr. Poul Jensen (gift)	Paleologu et al., 2009	DB
LB509 (human)	BioLegend	807707	DB
MJFR-14-6-4-2 (MJFR14, conformation-specific)	Abcam	Ab209538	DB
HuSyn1 (human)	Dr. Michael K. Lee	Lee et al., 2002	WB
Syn33 (conformation-specific oligomers)	Dr. Rakez Kayed (gift)	Sengupta et al., 2015	DB
**Tau Species**	**Company**	**Reference**	**Use**
Tau5 (total)	Millipore	MAB361	WB
AT8 (phospho-Ser-202 and -Thr-205)	Invitrogen	MN2010	WB
A11 (tau and αS oligomeric species)	Dr. Rakez Kayed (gift)	Khan et al., 2018	DB
CP13 (phospho-Ser-202)	Dr. Peter Davies (gift)	Khan et al., 2018	WB
OC (tau and αS amyloid fibrils)	Dr. Rakez Kayed (gift)	Khan et al., 2018	DB
PHF1 (phospho-Ser-396 and -Ser-404)	Dr. Peter Davies (gift)	Khan et al., 2018	WB
T22 (tau oligomers)	Dr. Rakez Kayed (gift)	Lasagna-Reeves et al., 2012	DB
**Neuron and Synapse Markers**	**Company**	**Reference**	**Use**
β-Synuclein	Thermo Fisher	PA5-25738	WB
MAP2	Novus Biologicals	NB300-213	IF
NeuN	Abcam	Ab177487	IF
PSD95	Abcam	Ab2723	IF
PSD95	Cell Signaling	3450	IF, WB
Synapsin 1/2	Synaptic Systems	106-002	WB
Synaptophysin	Abcam	Ab32127	IF, WB
**Glutamate Receptors**	**Company**	**Reference**	**Use**
GluA1 (D4N9V)	Cell Signaling	13185	WB
GluA2/3	Millipore	07-598	WB
GluN1	BD Pharmingen	556308	WB
GluN2A	Cell Signaling	4205	WB
**PrP-Fyn-GluN2B**	**Company**	**Reference**	**Use**
Fyn (FYN-01)	Abcam	Ab1187	WB
pTyr417-Src	Cell Signaling	2101	WB
GluN2B (D15B3)	Cell Signaling	4212	WB
pTyr1472-GluN2B	Cell Signaling	4208	WB
PrP (6D11)	BioLegend	808004	WB
PrP (8B4)	Santa Cruz	sc-47729	WB
**Network Markers**	**Company**	**Reference**	**Use**
Calbindin (D1I4Q)	Cell Signaling	13176	IF
c-Fos	Synaptic Systems	226-003	IF
Neuropeptide Y (D75YA)	Cell Signaling	11976	IF

Suppl. Table 1 – List of antibodies utilized in experiments. DB: Dot blot. IF: Immunofluorescence. WB: Western blot.

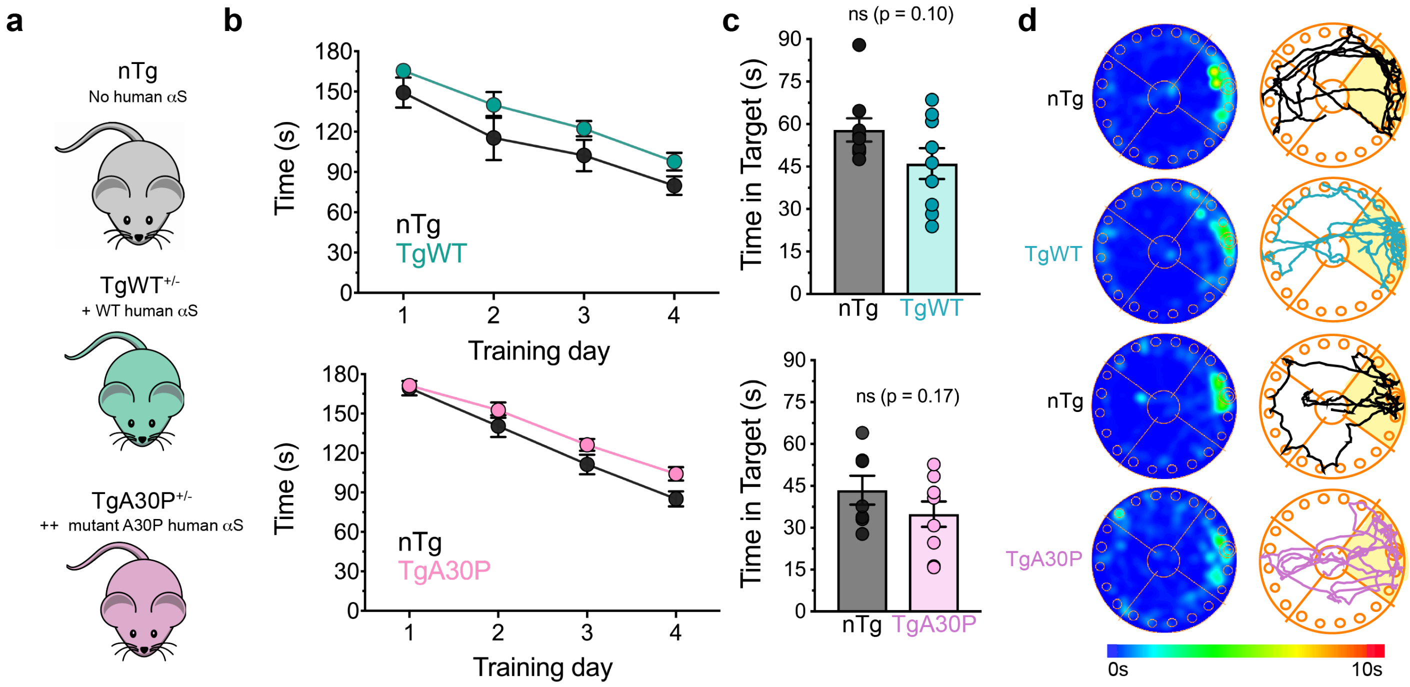

Suppl. Figure 1 – Aged TgWT and TgA30P mice do not display deficits in long-term spatial learning and memory via Barnes Maze. a. Depiction of animals used: wild-type animals (nTg) do not express human αS. TgWT animals are heterozygous for a transgene that expresses wild-type human αS. TgA30P animals are heterozygous a transgene that expressed human mutant A30P αS. **b.** Barnes maze (BM) training trials for 12-month-old nTg, TgA30P, and TgWT animals demonstrating duration of training trials per group during each of the four training days. TgWT training: two-way repeated measures ANOVA with Geisser-Greenhouse correction and Sidak's posthoc analysis revealed a significant effect of training ($F_{(2.545,40.72)} = 36.09$, $p < 0.0001$), no effect of $h\alpha S^{WT}$ genotype ($F_{(1,16)} = 3.249$, $p = 0.0903$), and no significant training day* $h\alpha S^{WT}$ interaction ($F_{(3,48)} = 0.1449$, $p = 0.9325$). TgA30P training: two-way repeated measures ANOVA with Geisser-Greenhouse correction and Sidak's posthoc analysis revealed a significant effect of training ($F_{(1.838,29.40)} = 143.9$, $p < 0.0001$), no effect of $h\alpha S^{A30P}$ genotype ($F_{(1,16)} =2.806$, $p = 0.1113$), and no significant training day* $h\alpha S^{A30P}$ interaction ($F_{(3,48)} = 1.645$, $p = 0.1915$). **c.** Time (seconds) spent occupying the target (goal) quadrant of the Barnes maze during the probe trial. Unpaired t test with Welch's correction. TgWT: t = 1.472, df = 14.91; TgA30P: t = 1.439, df = 15. TgWT: N = 9 animals/genotype. TgA30P: $n_{nTg} = 7$; $n_{TgA30P} = 9$. **d.** Left column: probe test BM occupancy heat maps obtained by averaging the location of all animals in each genotype and cohort. Right column: representative individual animal traces tracking movement during the BM probe test. BM orientation shown in Fig 1c, yellow shading indicates goal quadrant. The findings here demonstrate that postsynaptic deficits, as observed in TgA53T mice but not in TgWT or TgA30P, are required for cognitive decline by 12 months of age as TgWT and TgA30P do not display such deficits at this time point. ns: not significant. Error bars represent mean ± standard error of the mean (S.E.M).

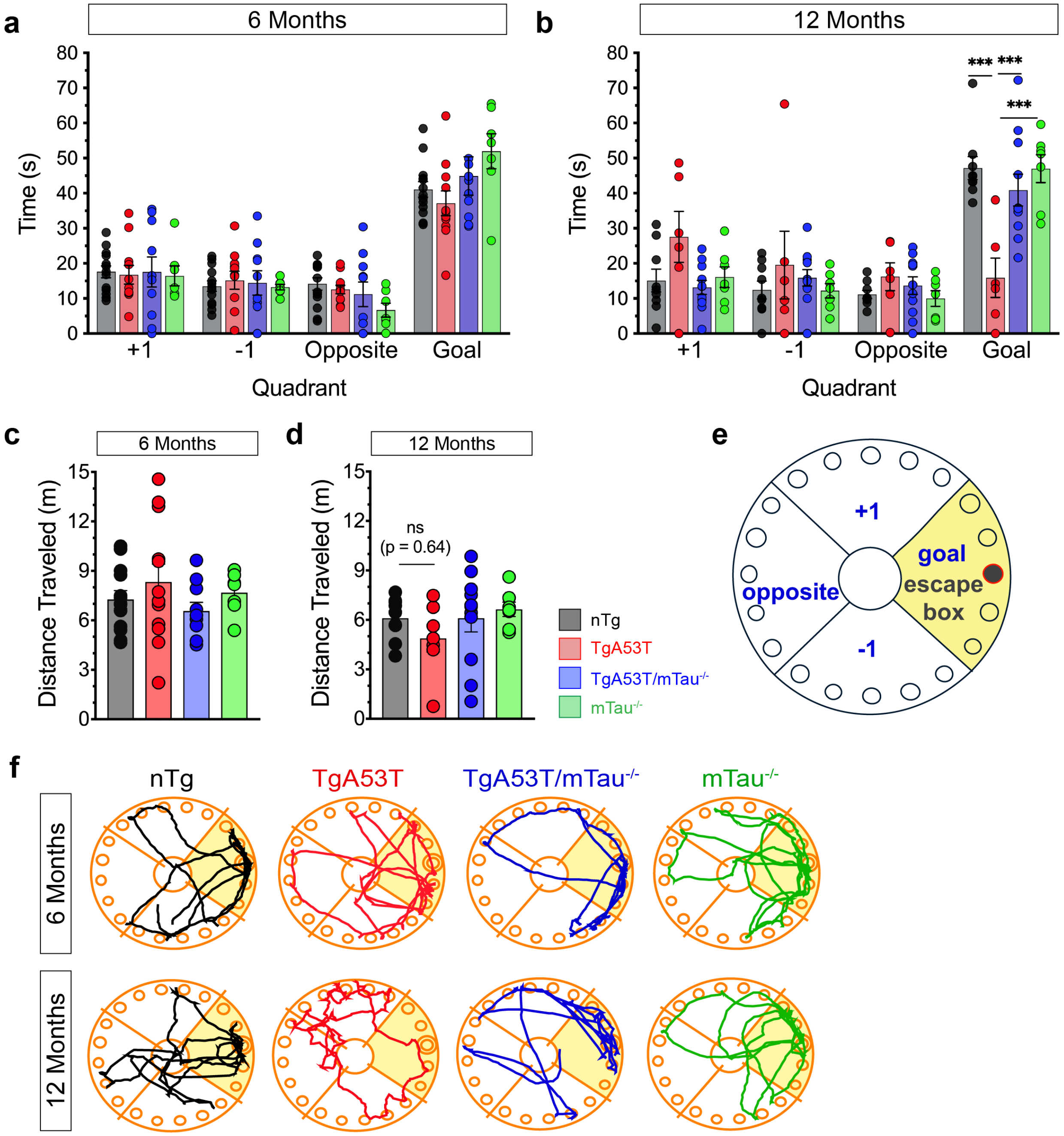

Suppl. Figure 2 – Barnes Maze quadrant analysis demonstrating progressive deficits in long-term spatial learning and memory in TgA53T mice in the absence of overt locomotor deficits. a and b. Analysis of time animals spent in each quadrant of the Barnes Maze (BM) during the probe test of the BM at 6 months (6M) **(a)** and 12 months (12M) **(b)**. 12M BM goal quadrant: $F_{(3,29)} = 10.47$, $p < 0.0001$, one-way ANOVA with Tukey's posthoc analysis. **c and d**. Total distance traveled on the BM by animals during the probe test at 6M **(c)** and 12M **(d)**. 12M: $F_{(3,31)} = 0.9377$, $p = 0.4347$ by one-way ANOVA with Tukey's posthoc analysis. 6M: $n_{nTg} = 13$; $n_{TgA53T} = 11$; $n_{TgA53T/mTau-/-} = 10$; $n_{mTau-/-} = 8$. 12M: $n_{nTg} = 9$; $n_{TgA53T} = 7$; $n_{TgA53T/mTau-/-} = 11$; $n_{mTau-/-} = 8$. **e.** BM diagram for testing and probe trial, with yellow shading indicating goal quadrant and dark grey showing escape box location in that quadrant. **f.** Representative individual animal traces tracking movement during the BM probe test. Yellow shading indicates goal quadrant. These results demonstrate that TgA53T mice have age-dependent deficits in spatial learning and memory that are dependent on endogenous mouse tau expression and precede onset of locomotor abnormalities. One-way ANOVA: *** $p < 0.001$. ns: not significant. Error bars represent mean ± S.E.M.

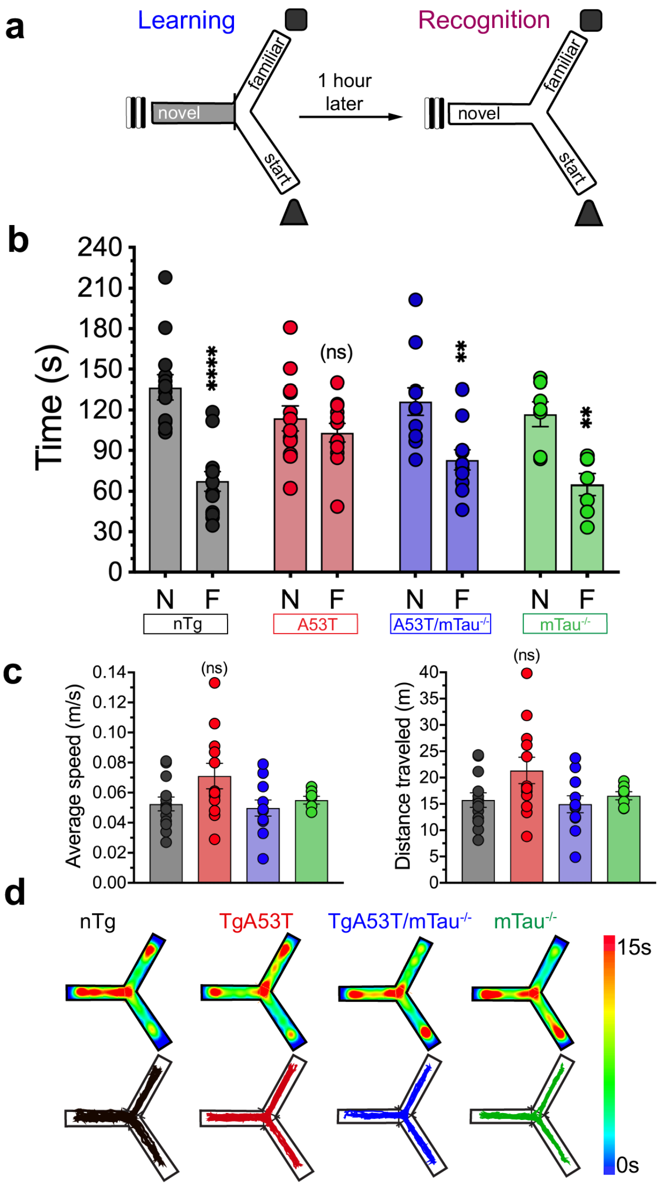

Suppl. Figure 3 – TgA53T mice show impaired short-term spatial learning and memory in a tau-dependent manner. a. Diagram depicting the Y maze (YM) orientation and experimental testing paradigm. YM testing was divided into two discrete phases: first, a Learning trial (Novel arm blocked off), followed by a Recognition test (all arms open, including Novel). Walls at the end of each arm were marked by different patterns: start with triangles, familiar with square checkerboard, and novel with stripes. **b.** Time spent in novel (N) and familiar (F) arms of the Y maze during the 300-second-long recognition trial. Unpaired t test with Welch's correction was used compare time animals spent in N versus F arms during recognition trial within genotype. nTg: $t = 5.967$, $df = 22$, $p < 0.0001$; TgA53T: $t = 0.9192$, $df = 20.40$, $p = 0.3687$; TgA53T/mTau$^{-/-}$: $t = 3.425$, $df = 18.34$, $p = 0.0030$; mTau$^{-/-}$: $t = 4.291$, $df = 11$, $p = 0.0011$. **c.** Left panel: average speed (meters/second) of mice during the recognition trial: $F_{(3,39)} = 2.609$, $p = 0.0652$, one-way ANOVA with Tukey's posthoc analysis. Right panel: total distance traveled (meters) by animals during the recognition trial. $F_{(3,39)} = 2.632$, $p = 0.0635$, one-way ANOVA with Tukey's posthoc analysis. **d.** Top row: heat maps demonstrating time all animals tested spent occupying areas of the YM during the recognition trial. Bottom row: individual trace of representative animal for each group tested during the recognition trial. Orientation of YM is preserved from diagram in **a**. Taken together, this experiment demonstrates that TgA53T mice present with tau-dependent deficits in spatial learning and memory via YM prior to developing deficits that can be detected via Barnes Maze. t test and one-way ANOVA: ** $p < 0.01$ and **** $p < 0.0001$. ns: not significant. Error bars represent mean ± S.E.M.

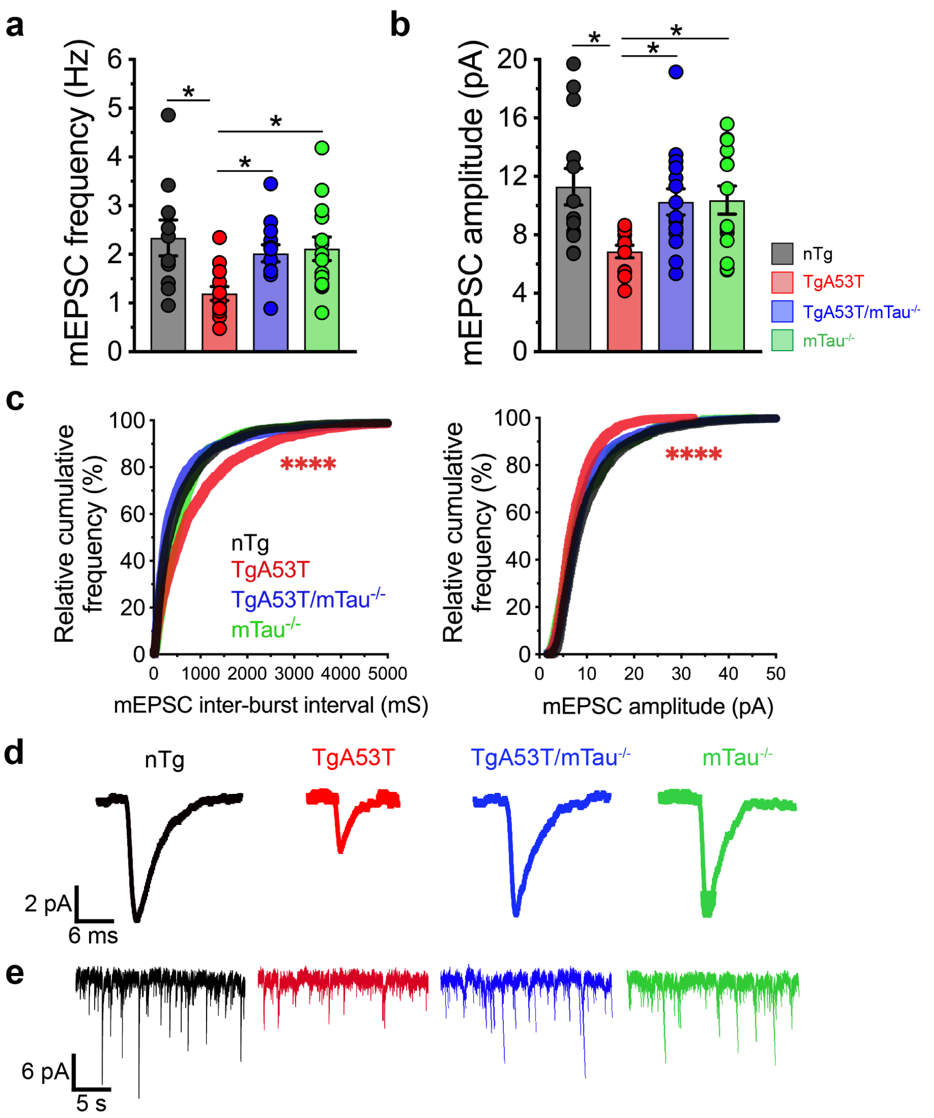

Suppl. Figure 4 – Tau is required for synaptic deficits in cultured TgA53T neurons. Average mini excitatory post-synaptic current (mEPSC) frequency **(a)** and amplitude **(b)** from *in vitro* recordings utilizing mouse primary hippocampal neuronal cultures. Each point represents average mEPSC frequency or amplitude from recording of a single neuron, with neurons coming from multiple litters (cultures). mEPSC frequency: $F_{(3,50)} = 3.872$, $p = 0.0144$, by one-way ANOVA and Tukey's posthoc analysis. mEPSC amplitude: $F_{(3,46)} = 4.551$, $p = 0.0071$, by one-way ANOVA and Tukey's posthoc analysis. **c.** Relative cumulative distribution frequency (CDF) of whole-cell mEPSC inter-burst interval (frequency, left panel) and amplitudes (right panel) from recordings of cultured neurons. mEPSC inter-burst interval CDF: Kolmogorov-Smirnov test, $D = 0.4681$, $p < 0.0001$. mEPSC amplitude CDF: Kolmogorov-Smirnov test, $D = 0.6042$, $p < 0.0001$. **d and e.** Representative traces of whole-cell mEPSCs recorded from dissociated mouse primary hippocampal neuronal cultures. mEPSC recordings from dissociated hippocampal neuron cultures demonstrate that both pre and postsynaptic deficits TgA53T neurons are tau-dependent. One-way ANOVA: * $p < 0.05$, **** $p < 0.0001$. Error bars represent mean ± S.E.M.

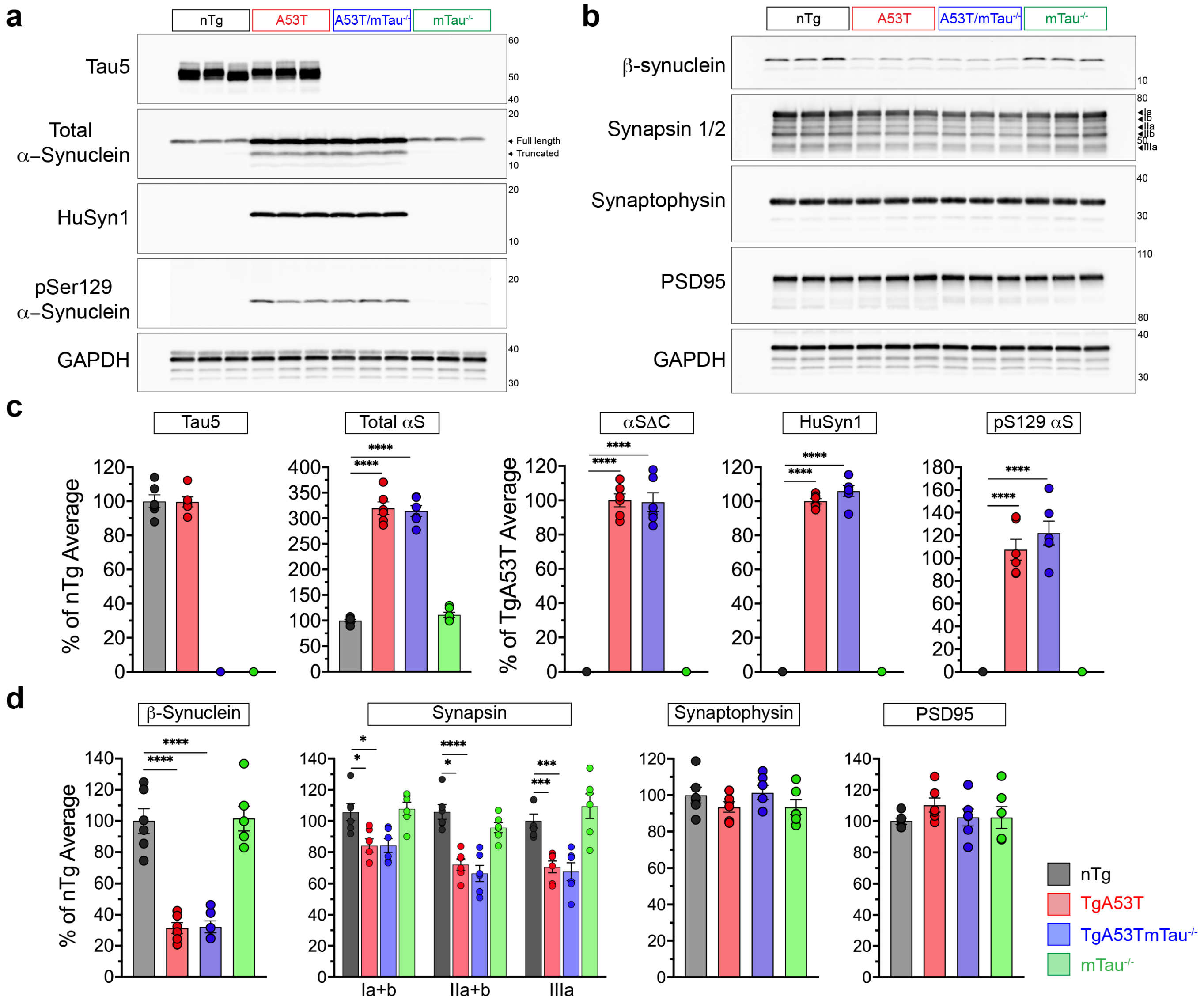

Suppl. Figure 5 – Cortical levels of αS expression, αS post-translational modifications, and synaptic proteins are tau-independent. a and b. Representative western blot analysis of cortical lysates from 12-month-old mice probing for total tau or αS **(a)**, or key pre and postsynaptic proteins of interest **(b)**. **c.** Densitometry of cortical protein expression. For tau and total αS, values were normalized to the average values for nTg samples within each gel. For truncated αS (αSΔC), HuSyn1 (human αS), αS phosphorylated at Ser129 (pSer-129-αS, pS129 αS), values were normalized to the average densitometric values of TgA53T samples within each gel. For all densitometry: one-way ANOVA with Tukey's posthoc analysis. Total αS: $F_{(3,20)} = 198.9$, $p < 0.0001$. αSΔC: $F_{(3,20)} = 298.2$, $p < 0.0001$. HuSyn1: $F_{(3,20)} = 1163.0$, $p < 0.0001$. pS129 αS: $F_{(3,20)} = 91.72$, $p < 0.0001$. **d.** Synapse densitometry, with values normalized GAPDH and compared to average of nTg samples. For western blot densitometry: one-way ANOVA with Tukey's posthoc analysis. β-synuclein: $F_{(3,20)} = 41.07$, $p < 0.0001$. Synapsin Ia+b: $F_{(3,20)} = 9.161$, $p = 0.0005$. Synapsin IIa+b. $F_{(3,20)} = 9.794$, $p = 0.0003$. Synapsin IIIa: $F_{(3,20)} = 13.70$, $p < 0.0001$. N = 6 animals/genotype. Densitometry shows that while β-synuclein and synapsin isoforms are decreased in TgA53T mice, they are not altered by loss of tau expression. Further, the levels of synaptophysin and PSD95 are comparable in all animals, indicating a lack of synaptic loss. One-way ANOVA: * $p < 0.05$, *** $p < 0.001$, and **** $p < 0.0001$. Error bars represent mean ± S.E.M.

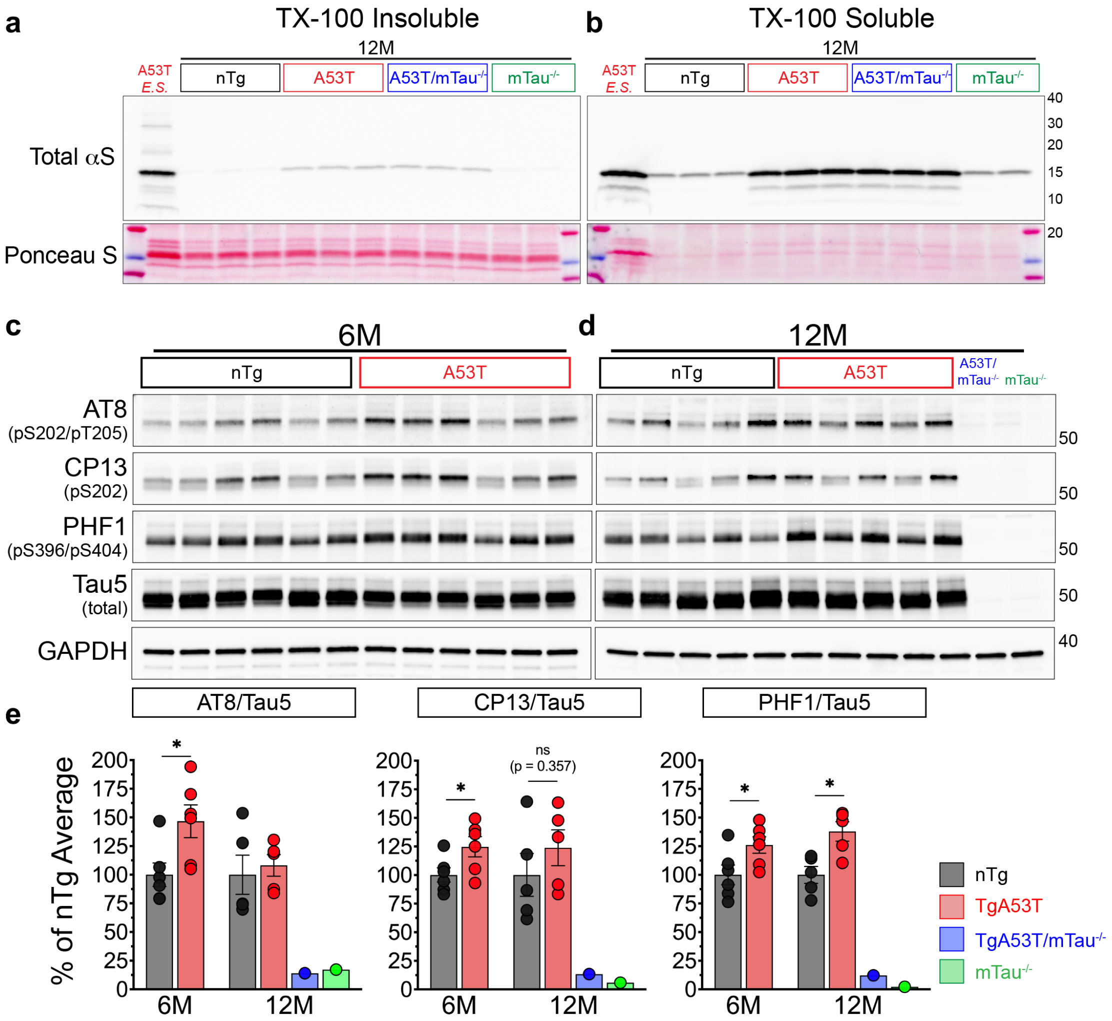

Suppl. Figure 6 – Analysis of αS solubility and tau phosphorylation in TgA53T mice. a and b. hαS^{A53T}-mediated, tau-mediated synaptic and cognitive dysfunction occurs in the absence of overt changes in insoluble αS species. Nonionic detergent (Triton™ X-100, TX-100) fractionation of insoluble and soluble components from 12-month-old (12M) mice. Representative western blot images for levels and species of total αS from the detergent-insoluble **(a)** and soluble fractions **(b).** Ponceau S staining of nitrocellulose membranes post-transfer demonstrates equal protein loading and consistent transfer across all lysates. The accumulation of nonionic detergent-insoluble αS species occurs exclusively in pathologically-affected brain regions of symptomatic "end-stage" (E.S.) TgA53T animals, particularly the brain stem and spinal cord. As a positive control, the *A53T E.S.* soluble and insoluble lysate fractions were prepared from the brain stem of an end stage TgA53T animal. Given the modest and comparable levels of insoluble αS in asymptomatic 12M TgA53T and TgA53T/mTau$^{-/-}$ animals, as well as the comparable levels of soluble αS levels in these groups, it suggests that soluble pathogenic species of αS are likely mediators of the hαS^{A53T}-driven synaptic and memory deficits. ($n_{TgA53T\text{-}E.S.}$ = 1, n_{nTg} = 3, n_{TgA53T} = 3, $n_{TgA53T/mTau-/-}$ = 3, $n_{mTau-/-}$ = 2). **c and d.** Synaptic and cognitive dysfunction in TgA53T are associated with modest but significant increases in pathological phosphorylated species of tau. Representative western blot analysis of hippocampal lysates from 6-month-old (6M) **(c)** 12-month-old (12M) **(d)** mice probing for disease-associated phosphorylated species of tau (AT8, CP13, and PHF1). **e.** Densitometry quantifying phosphorylated tau protein expression in the hippocampus. For 6M and 12M densitometry, expression of these pathological species of tau was normalized to total tau (Tau5) levels within each hippocampi, and then analyzed by unpaired t test with Welch's correction between nTg and TgA53T lysate values. $AT8_{6M}$: t = 2.656, df = 9.115, p = 0.260. $CP13_{6M}$: t = 2.286, df = 9.050, p = 0.0479. $PHF1_{6M}$: t = 2.340, df = 9.659, p = 0.0422. 6M: n_{nTg} = 6; $n_{TgAA53T}$ = 6. $AT8_{12M}$: t = 0.4183, df = 6.199, p = 0.06899. $CP13_{12M}$: t = 0.9803, df = 7.750, p = 0.3566. $PHF1_{12M}$: t = 23.353, df = 7.844, p = 0.0103. 12M: n_{nTg} = 5; $n_{TgAA53T}$ = 5; $n_{TgA53T/mTau-/-}$ = 1; $n_{mTau-/-}$ = 1. t test: * $p < 0.05$. ns: not significant. Error bars represent mean ± standard error of the mean (S.E.M).

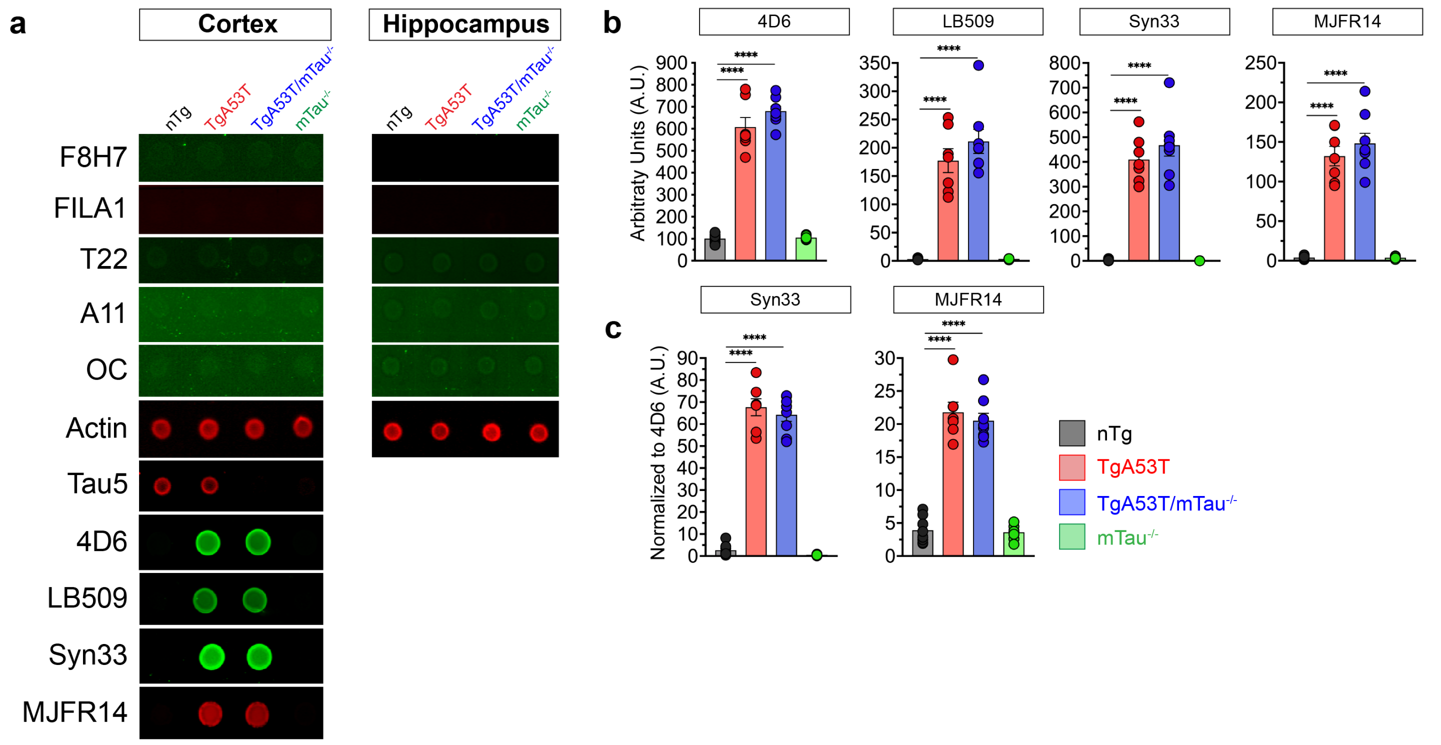

Suppl. Figure 7 – Synaptic and cognitive deficits in TgA53T mice are independent of expression or oligomeric-specific changes in αS or tau. a. Representative dot blots of species of αS and tau in non-denatured cortical and hippocampal lysates from 12-month-old mice (12M). The antibodies used here recognize epitopes associated with total αS (4D6), human αS (LB509), various pathological αS oligomers (F8H7, FILA1, Syn33, MJFR14), total tau (Tau5), pathological tau oligomers (T22), pan soluble oligomers (A11), and amyloid fibrils (OC). Antibody details can be found in Suppl. Table 1 (Online Resources). **b.** Densitometry of cortical protein expression normalized to the average densitometric values of nTg samples within each age. Only epitopes showing significant signal or increases in TgA53T mice were analyzed. For dot blot densitometry: one-way ANOVA with Tukey's posthoc analysis. 4D6: $F_{(3,27)} = 191.0$, $p < 0.0001$. LB509: $F_{(3,27)} = 59.12$, $p < 0.0001$. Syn33: $F_{(3,27)} = 86.28$, $p < 0.0001$. MJFR14: $F_{(3,27)} = 84.98$, $p < 0.0001$. **c.** Densitometry quantifying higher order synuclein species expression (Syn33 and MJFR14), normalized to the average total αS (4D6) within genotype. For dot blot densitometry: one-way ANOVA with Tukey's posthoc analysis: Syn33/4D6: $F_{(3,27)} = 246.4$, $p < 0.0001$. MJFR14/4D6: $F_{(3,27)} = 101.1$, $p < 0.0001$. N = 8 animals/genotype. Western blotting and densitometry demonstrates that synaptic and memory abnormalities in TgA53T mice are not due to changes in insolubility of αS or protein expression of αS but may occur downstream or independent of pathological αS species, in a tau-dependent manner. One-way ANOVA: **** $p < 0.0001$. Error bars represent mean ± S.E.M.

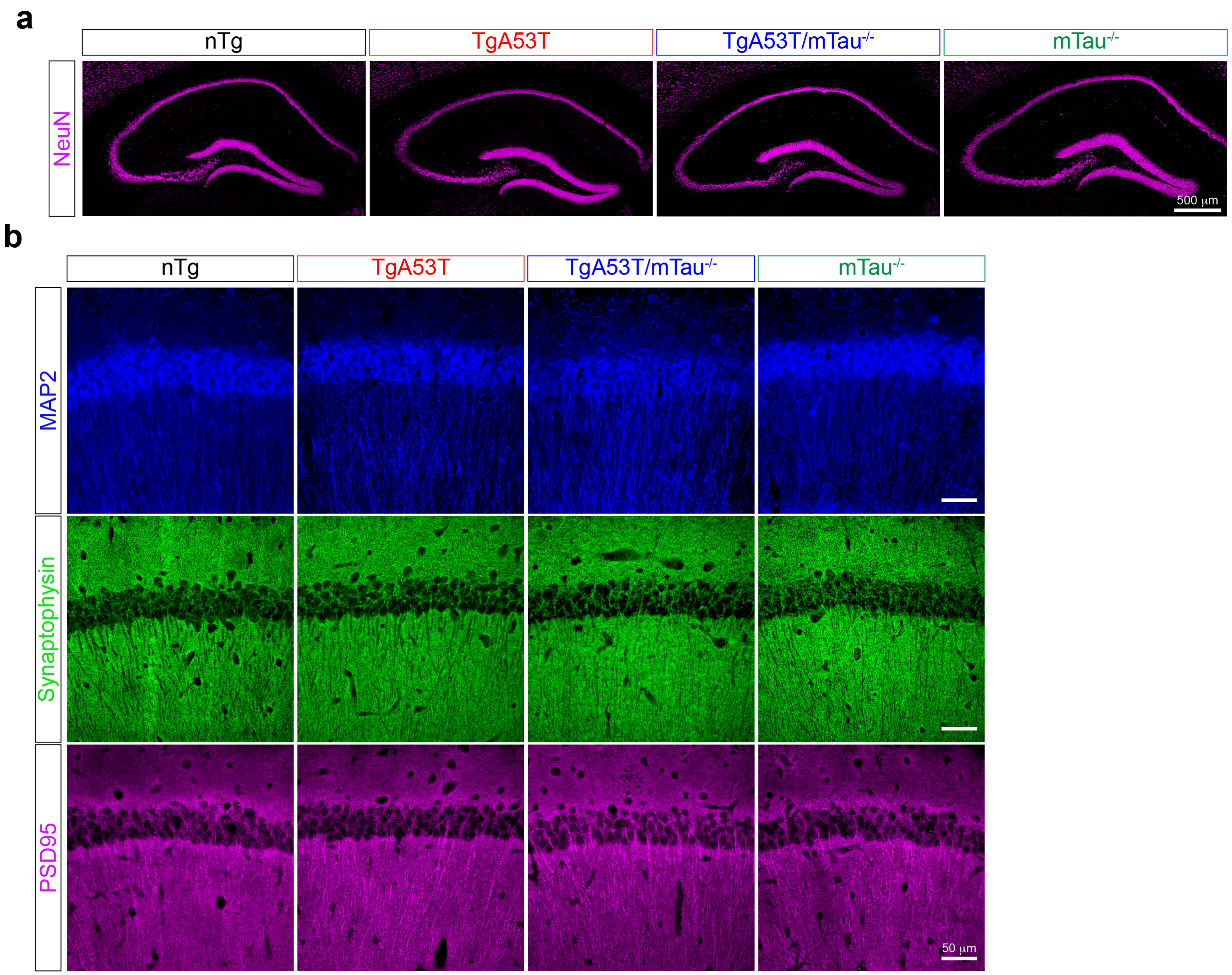

Suppl. Figure 8 – Neither synapse nor neuronal loss are required for synaptic dysfunction and memory deficits in TgA53T mice. a and b. Confocal images of immunohistochemical staining on 12-month-old hippocampi from nTg, TgA53T, TgA53T/mTau$^{-/-}$, and mTau$^{-/-}$ mice. Hippocampi stained for the neuronal nuclei marker NeuN **(a)**. CA1 region of the hippocampus stained for somatodendritic (MAP2), presynaptic (synaptophysin), and postsynaptic (PSD95) structures **(b).** Qualitatively, the lack of changes in gross hippocampal structure as well as synapse integrity when comparing nTg and TgA53T mice at 12 months of age indicates that synaptic dysfunction, rather than loss, is responsible for hαS^{A53T}-mediated cognitive decline.

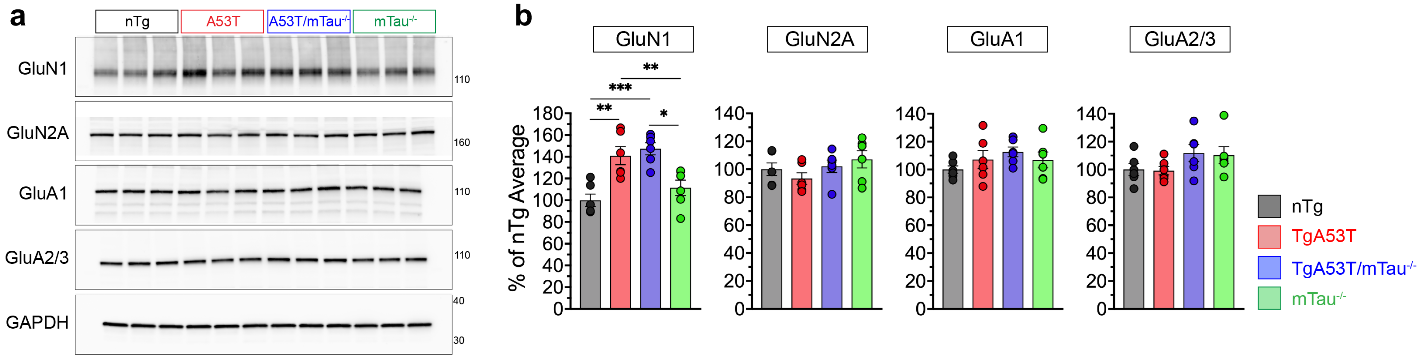

Suppl. Figure 9 – Tau-dependent biochemical changes in postsynaptic glutamatergic (AMPA and NMDA) receptors are region-specific. a. Representative western blot images for AMPAR (GluA) and NMDAR (GluN) subunits of cortical lysates from 12-month-old animals. **b.** Densitometry quantifying protein expression in isolated cortices. For all, values were normalized to the average densitometric values of nTg samples within each gel. For all densitometry: one-way ANOVA with Tukey's posthoc analysis. GluN1: $F_{(3,20)} = 11.49$, $p < 0.0001$. Compared to nTg mice cortices, cortical AMPA and NMDA receptor expression is not decreased in TgA53T mice at 12 months of age, when TgA53T mice have established synaptic deficits, evidence of network abnormalities, and memory loss. One-way ANOVA: * $p < 0.05$, ** $p < 0.01$, and *** $p < 0.001$. Error bars represent mean ± S.E.M.

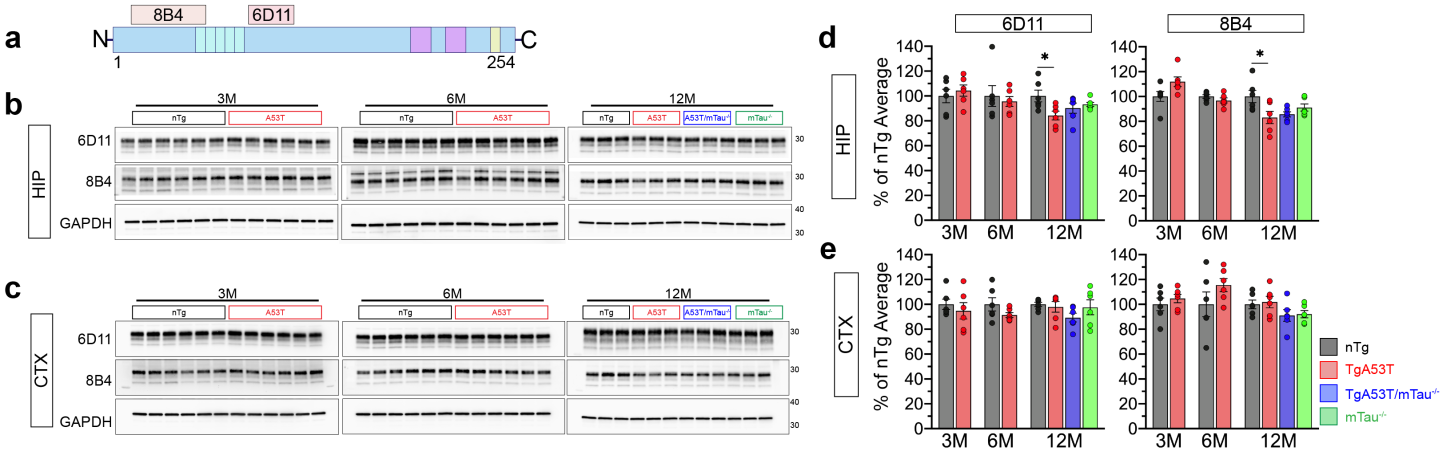

Suppl. Figure 10 – Tau-dependent synaptic dysfunction in TgA53T neurons is not associated with increases in PrP expression and signaling. a. Graphical depiction of cellular prion protein (PrP^C) primary structure and antibody binding regions. **b and c.** Representative western blot images of hippocampal **(b)** and cortical **(c)** lysates for PrP^C expression at 3, 6 and 12 months of age (3M, 6M, and 12M, respectively). **d and e.** Densitometry of protein expression in hippocampal (HIP) **(d)** and cortical (CTX) **(e)** lysates, normalized to the average densitometric values of nTg samples within each gel. For 3M and 6M densitometry, unpaired t test with Welch's correction. For 12M densitometry: one-way ANOVA with Tukey's posthoc analysis. 12M HIP 6D11: $F_{(3,20)} = 3.193$, $p = 0.0458$. 12M HIP 8B4: $F_{(3,20)} = 3.685$, $p = 0.0292$. These results demonstrate that activation of PrP^C signaling is not required for the age-dependent synaptic and memory deficits in TgA5T mice. t test and one-way ANOVA: * $p < 0.05$. Error bars represent mean ± S.E.M.

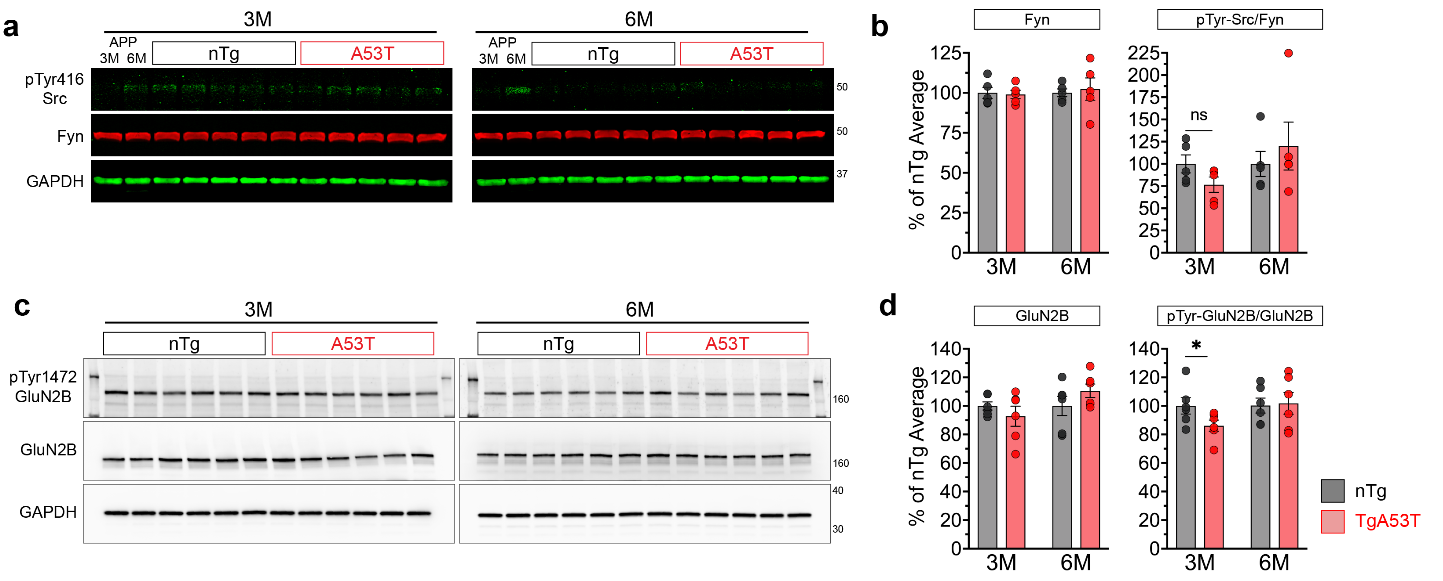

Suppl. Figure 11 – Tau-mediated, mutant αS-induced synaptic and cognitive deficits are independent of postsynaptic Fyn and GluN2B activation. a and b. Western blot images for activation status of both Fyn (via pTyr416 Src) **(a)** and NMDA receptor subunit GluN2B (via pTyr1472 GluN2B) **(c)** 3 (left) and 6 (right) months of age (3M and 6M, respectively) through analysis of total and active phosphorylated states. **b and d.** Densitometry quantifying 3M and 6M cortical lysates, normalized to the average densitometric values of nTg samples within each gel. Activation status was determined through examining ratio of values of activating phosphorylation site: pTyr416 for Fyn **(b)** and pTyr1472 for GluN2B **(d)**, normalized to respective total levels of protein. For 3M and 6M densitometry, unpaired t test with Welch's correction. 3M pTyr-Src/Fyn: $t = 1.761$, $df = 8$, $p = 0.1163$. 3M pTyr-GluN2B/GluN2B: $t = 2.539$, $df = 8.066$, $p = 0.0345$. These findings build on Suppl. Figure 9, further showing that TgA53T mice do not display increased activation of the PrP^{C}-Fyn-GluN2B signaling cascade as compared to age-matched nTg littermates. Together, these results suggest that $h\alpha S^{A53T}$-driven and impairments requiring tau are mediated independent of this pathway. t test: * $p < 0.05$. ns: not significant. Error bars represent mean ± S.E.M.

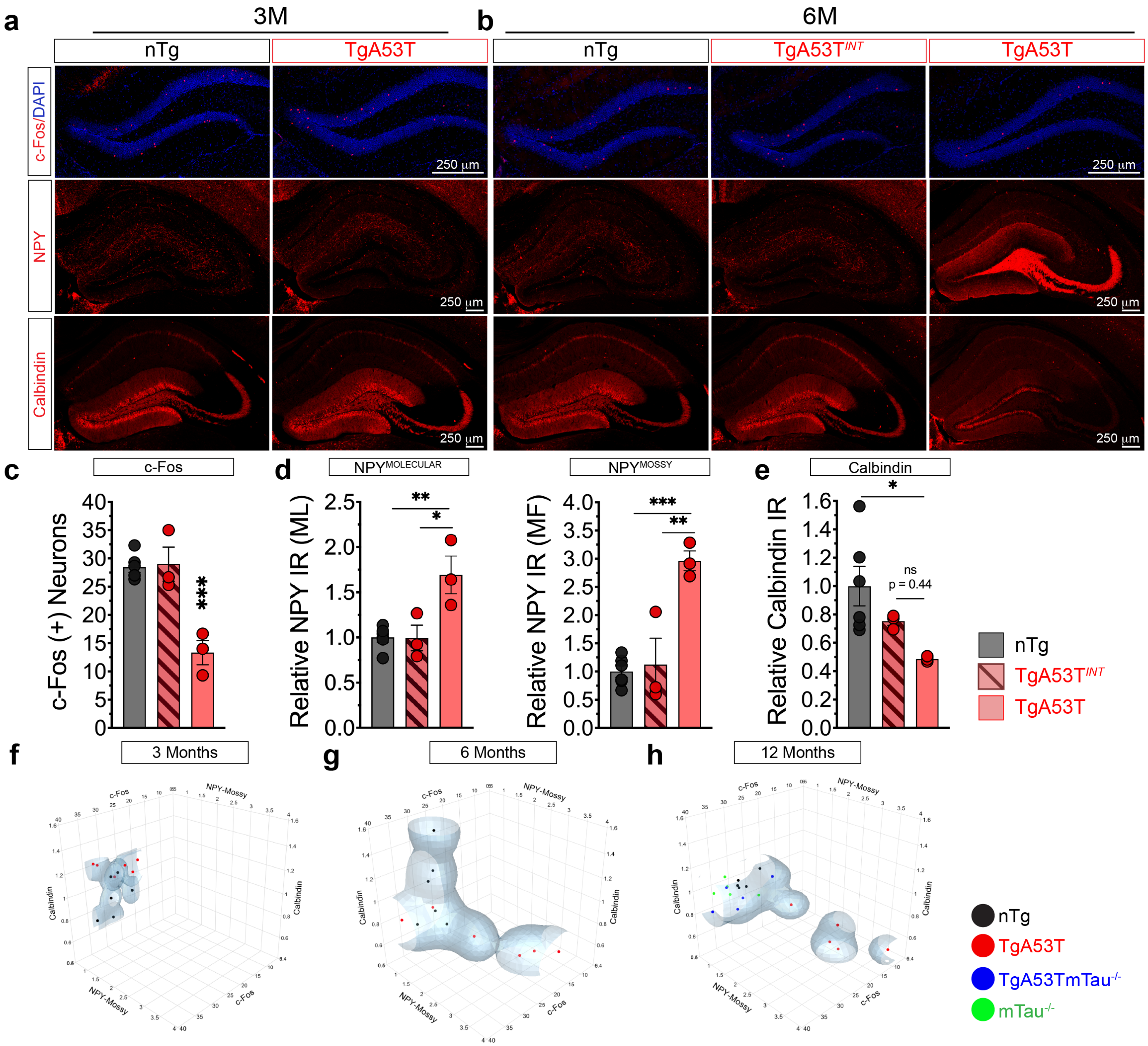

Suppl. Figure 12 – Activity-dependent remodeling of hippocampal circuits is not observed in younger TgA53T mice with intact cognition and appears to follow glutamatergic signaling deficits. a and b. Representative confocal images from dentate gyri and hippocampi of 3-month-old (3M, **a**) and 6-month-old (6M, **b**) nTg and TgA53T mice stained for c-Fos, NPY, and calbindin. 3M and 6M c-Fos, NPY, and calbindin quantification values are reported in Fig 9c-e. c-Fos scale bar: 300 µm. NPY and calbindin scale bar: 250 µm. **c-e.** Quantification of immunoreactivity (IR) via cell counting (**c:** c-Fos) or densitometry (**d:** NPY in the Molecular Layer "Molecular", ML; NPY in the Mossy Fiber pathway, "Mossy", MF; and, **e:** calbindin) at 6M. 6M TgA53T animals were classified into two groups as some TgA53T mice displayed activity-related changes in c-Fos, calbindin, and NPY while others, the TgA53T^{INT} (for "Intermediate") resembled their age-matched nTg littermate controls. c-Fos: $F_{(2,9)}$= 22.16, $p = 0.0003$; NPY-Molecular: $F_{(2,9)} = 10.60$, $p = 0.0043$; NPY-Mossy: $F_{(2,9)} = 20.74$, $p = 0.0004$; Calbindin: $F_{(2,9)} = 4.174$, $p = 0.0522$. All by one-way ANOVA with Tukey's posthoc analysis. $n_{nTg} = 6$; $n_{TgA53T} = 3$; $n_{TgA53T\text{-}INT} = 3$. **f-h.** Three-dimensional (3D) X-Y-Z scatterplot of c-Fos, NPY (Mossy Fiber, "NPY-Mossy"), and calbindin immunostaining at 3 **(f)**, 6 **(g)**, and 12 **(h)** months (3M, 6M, and 12M, respectively). 3M TgA53T mice do not display evidence of network remodeling. The two distinct TgA53T populations present at 6M are clearly observed: one TgA53T group displayed activity-related changes in c-Fos, calbindin, and NPY while others, the TgA53T^{INT} (for "Intermediate") more closely resembled their age-matched nTg littermate controls. By 12M, all aged TgA53T mice demonstrate signs of chronic hippocampal network hyperactivity. One-way ANOVA: * $p < 0.05$, ** $p < 0.01$, and *** $p < 0.001$. ns: not significant. Error bars represent mean ± S.E.M.

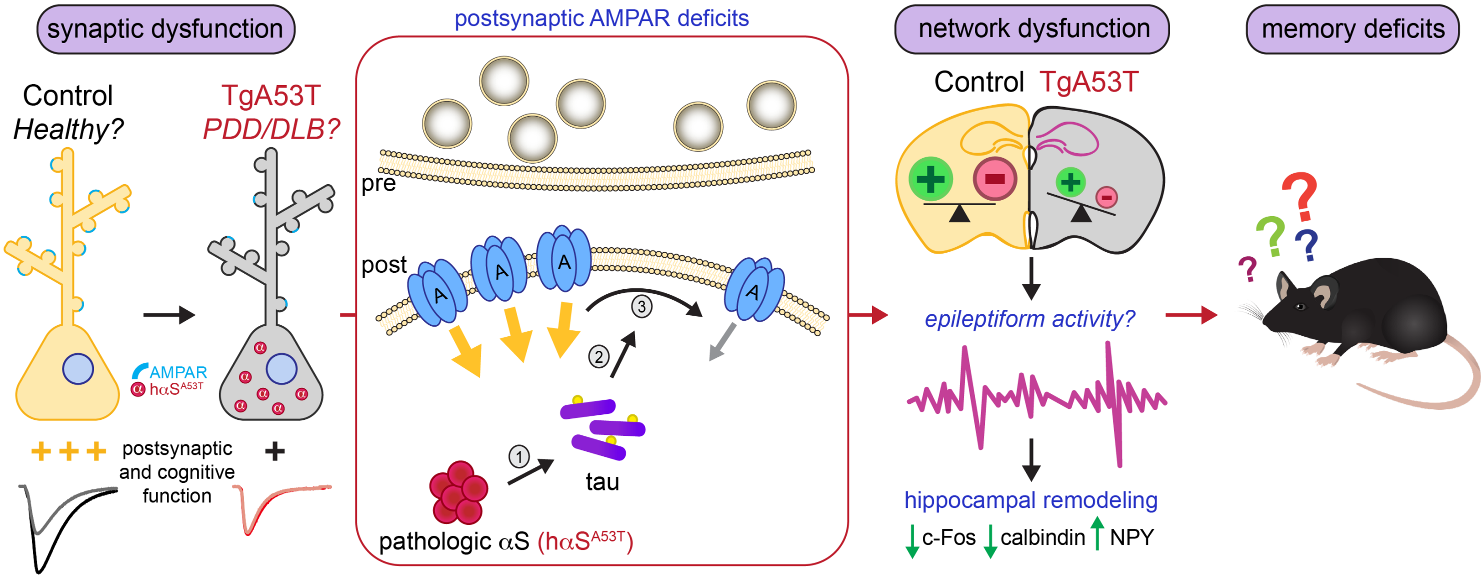

Suppl. Figure 13 – Proposed model for αS-mediated, tau-dependent synaptic and cognitive deficits in hαS^{A53T} mice. Neurons from cognitively-intact nTg control mice display intact AMPA receptor (AMPAR) expression and function, and thus display intact synaptic transmission. The presence of pathological species of αS, such as hαS^{A53T} in TgA53T mice leads to progressive postsynaptic synaptic deficits that underlie memory deficits in this model, and potentially PDD or DLB patients, without overt structural alterations at the synaptic or neuronal level. Pathogenic αS (hαS^{A53T}) expression or accumulation (1) leads to tau phosphorylation and mislocalization to dendritic spines (2), producing physiological deficits in AMPAR -mediated signaling and synaptic plasticity, likely through reductions in AMPAR expression (3). These hαS^{A53T}-mediated AMPAR deficits culminate in reduced glutamatergic signaling, particularly at hippocampal pyramidal neuron synapses. The AMPAR deficits lead to more global, circuit-level abnormalities in the brains of TgA53T mice. For example, it is possible that hαS^{A53T}-mediated, tau-dependent synaptic depression may also affect inhibitory interneurons, leading to homeostatic imbalance between inhibitory and excitatory neurotransmission and aberrant excitatory activity. These epileptiform changes can then lead to homeostatic responses in hippocampal circuits that attempt to suppress this hyperactivity, representing a potential mechanism underlying the hippocampal remodeling observed in the brains of TgA53T mice. However, future studies are warranted to better elucidate the biochemical, physiological, and structural mechanisms connecting hαS^{A53T}-mediated synaptic dysfunction and aberrant network changes. Ultimately, we hypothesize that hαS^{A53T}-driven, tau-mediated abnormalities at the individual synapse lead to network-level perturbations that together contribute to and exacerbate memory deficits in TgA53T mice. PDD: Parkinson's disease dementia. DLB: dementia with Lewy bodies. Pre: presynaptic neuron. Post: postsynaptic neuron. A: AMPAR. α: hαS^{A53T}.
